# Promised and Lottery Airtime Incentives to Improve Interactive Voice Response Survey Participation Among Adults in Bangladesh and Uganda: Randomized Controlled Trial

**DOI:** 10.2196/36943

**Published:** 2022-05-09

**Authors:** Dustin Garrett Gibson, Gulam Muhammed Al Kibria, George William Pariyo, Saifuddin Ahmed, Joseph Ali, Alain Bernard Labrique, Iqbal Ansary Khan, Elizeus Rutebemberwa, Meerjady Sabrina Flora, Adnan Ali Hyder

**Affiliations:** 1 Johns Hopkins Bloomberg School of Public Health Baltimore, MD United States; 2 Institute of Epidemiology, Disease Control and Research Dhaka Bangladesh; 3 Makerere University School of Public Health, Makerere University College of Health Sciences Kampala Uganda; 4 Milken Institute School of Public Health, George Washington University Washington DC, MD United States

**Keywords:** mobile phone survey, interactive voice response survey, survey, interactive voice response, non-communicable disease, surveillance, airtime incentive, response rate, cooperation rate, communicable disease, Uganda, Bangladesh, low income, middle income, LMIC, Africa, incentive, RCT, randomized controlled trial, lottery, cooperation, participation

## Abstract

**Background:**

Increased mobile phone penetration allows the interviewing of respondents using interactive voice response surveys in low- and middle-income countries. However, there has been little investigation of the best type of incentive to obtain data from a representative sample in these countries.

**Objective:**

We assessed the effect of different airtime incentives options on cooperation and response rates of an interactive voice response survey in Bangladesh and Uganda.

**Methods:**

The open-label randomized controlled trial had three arms: (1) no incentive (control), (2) promised airtime incentive of 50 Bangladeshi Taka (US $0.60; 1 BDT is approximately equivalent to US $0.012) or 5000 Ugandan Shilling (US $1.35; 1 UGX is approximately equivalent to US $0.00028), and (3) lottery incentive (500 BDT and 100,000 UGX), in which the odds of winning were 1:20. Fully automated random-digit dialing was used to sample eligible participants aged ≥18 years. The risk ratios (RRs) with 95% confidence intervals for primary outcomes of response and cooperation rates were obtained using log-binomial regression.

**Results:**

Between June 14 and July 14, 2017, a total of 546,746 phone calls were made in Bangladesh, with 1165 complete interviews being conducted. Between March 26 and April 22, 2017, a total of 178,572 phone calls were made in Uganda, with 1248 complete interviews being conducted. Cooperation rates were significantly higher for the promised incentive (Bangladesh: 39.3%; RR 1.38, 95% CI 1.24-1.55, *P*<.001; Uganda: 59.9%; RR 1.47, 95% CI 1.33-1.62, *P*<.001) and the lottery incentive arms (Bangladesh: 36.6%; RR 1.28, 95% CI 1.15-1.45, *P*<.001; Uganda: 54.6%; RR 1.34, 95% CI 1.21-1.48, *P*<.001) than those for the control arm (Bangladesh: 28.4%; Uganda: 40.9%). Similarly, response rates were significantly higher for the promised incentive (Bangladesh: 26.5%%; RR 1.26, 95% CI 1.14-1.39, *P*<.001; Uganda: 41.2%; RR 1.27, 95% CI 1.16-1.39, *P*<.001) and lottery incentive arms (Bangladesh: 24.5%%; RR 1.17, 95% CI 1.06-1.29, *P*=.002; Uganda: 37.9%%; RR 1.17, 95% CI 1.06-1.29, *P*=.001) than those for the control arm (Bangladesh: 21.0%; Uganda: 32.4%).

**Conclusions:**

Promised or lottery airtime incentives improved survey participation and facilitated a large sample within a short period in 2 countries.

**Trial Registration:**

ClinicalTrials.gov NCT03773146; http://clinicaltrials.gov/ct2/show/NCT03773146

## Introduction

It is well evidenced that low- and middle-income countries are undergoing demographic and epidemiologic transitions; there is an increasing burden from noncommunicable diseases such as hypertension, diabetes, stroke, and other diseases [1,2]. Four mostly modifiable risk factors primarily contribute to this high noncommunicable disease burden—unhealthy diets, physical inactivity, tobacco use, and excessive alcohol consumption [3,4]. Continuous surveillance and monitoring of these risk factors are crucial to prevent and control noncommunicable diseases [5]. However, collecting data for noncommunicable disease risk factor surveillance is challenging in low- and middle-income countries due to the level of effort, time, and money required for face-to-face interviews and associated data management, analysis, and reporting [6].

High-income countries implement telephone interviews to obtain population-level estimates for health-related outcomes [7,8]. Participation in telephone surveys has declined in recent years in high-income countries, and other survey methods (eg, web-based) are also used to collect health-related data. Although most low- and middle-income countries do not have the infrastructure for conducting landline- or web-based surveys, increased access and ownership of mobile phones in low- and middle-income countries provide the opportunity to use mobile phone numbers for household surveys [9]. Throughout the COVID-19 pandemic, mobile phone surveys have been used to collect data on a broad range of topics [10-13].

There are several options for delivering mobile phone surveys: SMS text messaging, call center interviews by a human operator, and interactive voice response [14]. Interactive voice response is a mobile phone survey method wherein respondents use their mobile phone keypad to select answers from prespecified options (eg, “press 1 if you are male; press 2 if you are female”). Incentives for mobile phone surveys, often delivered as cash, coupons, vouchers, or airtime balances, have been shown to increase survey participation [15]. It could also be considered as compensation for the time spent by participants. Incentives may reduce the amount of time required for data collection by recruiting the optimum number of participants in a shorter time period. In high-income countries, where there is a larger body of evidence on a range of different survey types, providing an incentive beforehand typically produces better survey response than promised or lottery incentives across a [16-18]; however, overall findings have been mixed, and some studies [19,20] show that providing incentives does not improve participation. Past studies [21-24] from low- and middle-income countries have also shown similar mixed results. Studies [21,22] have also shown that delivering incentives to everyone, than using a lottery, can increase participation and reduce cost; there have been limited number of studies [23,24] examining the impact of different incentive amount on the overall survey cost, and investigating these factors would be helpful in understanding the feasibility of mobile phone surveys for future data collection. We aim to fill in these gaps in the literature by assessing the effect of different airtime incentive approaches on the cooperation, response, contact, and refusal rates of an interactive voice response survey for noncommunicable disease behavioral risk factors.

## Methods

### Study Design

We conducted a randomized controlled trial in Bangladesh (an area of approximately 148,000 km^2^ with an estimated population of 160 million [25]) and Uganda (an area of approximately 241,000 km^2^ with an estimated population of 40 million [25]). In 2017, mobile phone subscription rates were 83 and 55 subscribers per 100 people in Bangladesh and Uganda, respectively [9].

In this trial, incentives were delivered as airtime (ie, mobile phone balance). Participants were randomized to 1 of 3 study arms: no incentive (control arm), a promised airtime incentive of 50 Bangladeshi Taka (US $0.60; 1 BDT is approximately equivalent to US $0.012) or 5000 Ugandan Shilling (US $1.35; 1 UGX is approximately equivalent to US $0.00028) for completing the interactive voice response survey, or lottery incentive (500 BDT and 100,000 UGX), wherein the odds of winning were 1:20. The conduct, analysis, and reporting of results were performed in accordance with Consolidated Standards of Reporting Trials guidelines [26].

### Participants

Participants were sampled using a fully automated random-digit dialing method [27]. Briefly, the country codes along with the 3-digit sequence specific to the mobile network operator were used as the base for potential mobile phone numbers. The remaining 7 digits were generated randomly. Respondents who self-reported being aged 18 years or older were eligible for the trial. The survey was programmed to have a designated local number appear on the respondent’s caller ID screen.

### Randomization and Masking

The automated randomization process was performed within the interactive voice response platform to cover all mobile phone networks in both countries. Participants were randomized after selecting the survey language but prior to consent (Figure S1 in Multimedia Appendix 1). Due to the nature of the study design, participants were informed about the incentive during the survey introduction and, therefore, were not blinded to study arm allocation. Statisticians involved in data cleaning and analysis were blinded to participant allocation.

### Procedures

The overall procedures were similar in both countries. Interactive voice response surveys were sent only once to each phone number, and calls were made between 8 AM and 8 PM local time. Respondents who picked up the phone were instructed to select a language from a list of languages: Bangla or English in Bangladesh and Luganda, Luo, Runyakitara, or English in Uganda. Candidate participants listened to a description of the survey objectives and requirements for the incentive (ie, survey completion) as applicable (Table S1 and Figure S1 in Multimedia Appendix 1). Participants were told that they would not incur any expenses by answering the survey. Age-eligibility was confirmed (ie, “Are you 18 years or older? If yes, press 1; if no, press 3“). Age-eligible candidates were provided the consent disclosure statement and asked to authorize their participation by pressing the 1 button on the mobile phone. Participants answered demographic and noncommunicable disease questions, and only those who completed the survey received the incentive. Participants were instructed to press the star key to repeat any questions.

Demographic data on age, gender, education, and location were collected to perform subgroup analysis (ie, to identify differences in participation by those characteristics). Noncommunicable disease questions were grouped into 5 modules: tobacco use, alcohol consumption, dietary habits (including consumption of fruits, vegetables, and salt), physical activity, and medical conditions (including hypertension and diabetes). Because respondents could end the interview before finishing all modules, the order of the noncommunicable disease modules was randomized to minimize attrition and to ensure that data reporting errors were as randomly distributed as possible. Questions within a module were not randomized in order to maintain skip patterns. The questionnaire was adapted from standardized surveys [28], and initial cognitive testing and user groups were conducted with people who identified themselves as being from a low- and middle-income country at Johns Hopkins University [29]. A series of key informant interviews and focus group discussions were also conducted in each country to assess the comprehensibility and accuracy of translated questionnaires and to improve the usability of the interactive voice response platform.

### Ethical Approval

Johns Hopkins Bloomberg School of Public Health, Makerere University School of Public Health, The Uganda National Council for Science and Technology, and The Institute of Epidemiology Disease Control and Research institutional review boards approved the study protocol (number NCT03773146). The study was registered (NCT03773146), and the study protocol has been published elsewhere [30].

### Outcomes

The primary outcomes of this trial were response rates 4 and cooperation rates 1, as defined by the American Association for Public Opinion Research (Table S2 in Multimedia Appendix 1) [31]. Response rate calculations included partial and complete surveys in the numerator. Cooperation rate was calculated as the proportion of complete interviews from all eligible respondents, but the calculation did not include people who immediately hung up or who did not answer the age question in the denominator. Secondary outcomes were contact rate 2 and refusal rate 2 [31]. The cooperation rate was the number of complete interviews divided by the sum of complete, partial, and noninterviews. Complete interviews were defined as respondents who answered at least 4 of the 5 noncommunicable disease modules. Partial interviews were defined as respondents who answered between 1 and 3 noncommunicable disease modules. Noninterviews included refusals (ie, participants who ended the survey at the consent question) and break-offs (ie, participants who were 18 years or older but did not complete an noncommunicable disease module). The response rate was calculated as the number of complete and partial interviews divided by the total number of complete and partial interviews, refusals, break-offs, and the estimated proportion of age-eligibility unknown calls (individuals who initiated the survey but did not answer the age question). The estimated proportion of unknown eligibility was obtained from the proportion of participants who responded to the age-screening question and indicated they were 18 years or older. Calls were classified as ineligible if the individual indicated an age below 18 years or did not pick up the phone. As a secondary analysis, contact refusal and response rates were calculated without applying e for the unknown participants.

### Statistical Analysis

Demographic characteristics of complete interviews were described by study arms and compared using chi-square tests. Using the control arm as the reference category, risk ratios (RR) and 95% confidence intervals were calculated for contact, response, refusal, and cooperation rates with log-binomial regression [32]. To assess any potential effect modification of incentives on cooperation rates, the log-binomial models were extended and interaction terms with education, gender, age, and location were tested. We did not assess any effect modification for response rates because its equation included disposition codes for *Unknown* (participants who did not answer any of the demographic questions).

We calculated pooled risk ratios for different incentive arms using random-effects meta-analysis [33]. The heterogeneity statistic (ie, *I*^2^) was estimated using the Mantel-Haenszel method. The *I*^2^ statistic indicates the proportion of variability in effect that resulted from heterogeneity instead of chance or sampling error. A lower *I*^2^ statistic suggests lower heterogenicity. We calculated the direct delivery cost per complete survey, which included the cost of airtime used to deliver the survey and the incentive amount, as applicable. We summed the total call durations by arm and multiplied by per-minute airtime cost (US $0.04 in Bangladesh and $0.10 in Uganda) to produce the estimated cost per completed survey. Stata (version 14.0; StataCorp LLC) was used to analyze data. An α=.05 was assumed for all tests of statistical significance.

### Sample Size

We used the same assumptions to calculate required sample sizes for the trial in both countries. With a 30% cooperation rate of the control arm, 5% type 1 error, and 80% power, 376 participants were required to complete the interview for each study arm in order to detect a 10% difference between control and incentive arms. The total required sample size (ie, complete surveys) was 1128 in each country. As recommended [34], we did not inflate the sample for multiple comparisons.

## Results

**Figure 1:**
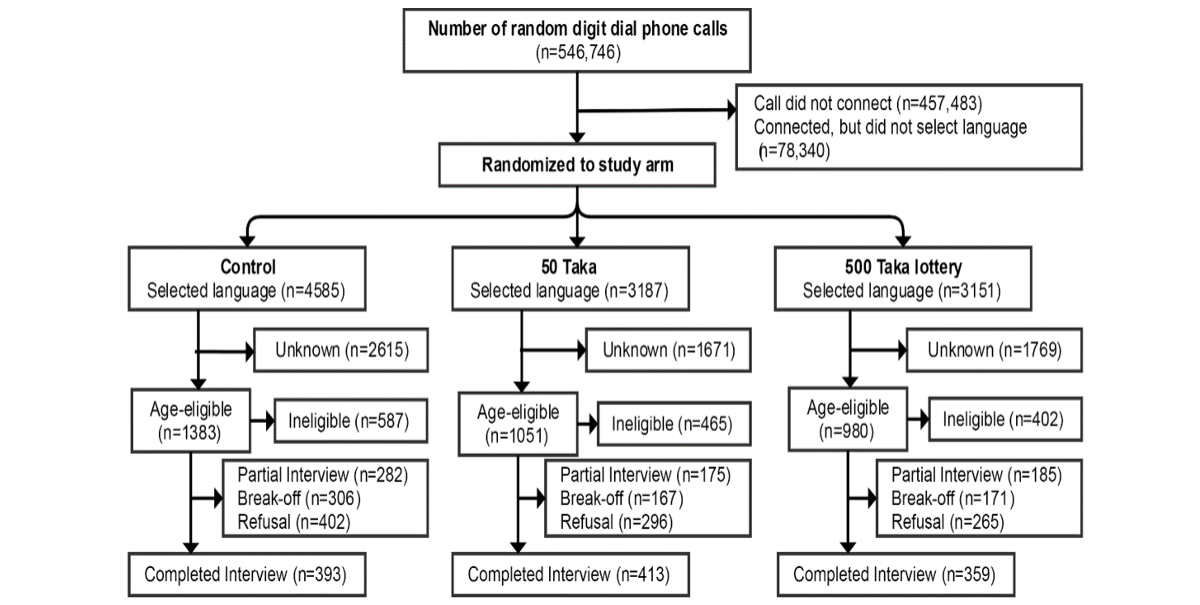
Consolidated Standard of Reporting Trial diagram of study participants in Bangladesh.

**Figure 2:**
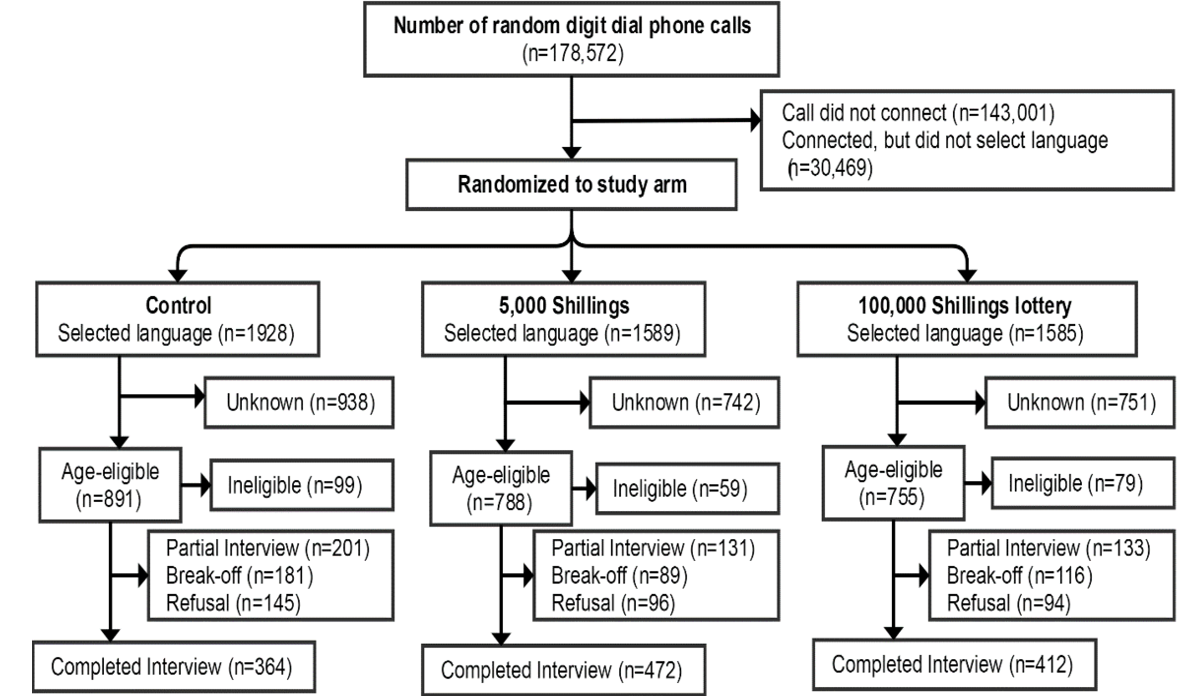
Consolidated Standard of Reporting Trial diagram of study participants in Uganda.

From June 14, 2017 to July 14, 2017, a total of 1165 compete interviews were obtained from 546,746 phone calls in Bangladesh (Figure 1). In Uganda, 178,572 calls were made between March 26 and April 22, 2017 to obtain 1248 complete interviews (Figure 2). In both countries, the sociodemographic characteristics of complete interviews were similar across study arms (Table 1). Of 1165 respondents in Bangladesh, 89.4% (n=1042) respondents were male. Of 1248 respondents in Uganda, 76.0% (n=948) respondents were male. Most respondents were between the ages of 18 to 29 years old—74.4% (867/1165) and 71.0% (886/1248) in Bangladesh and Uganda, respectively.

**Table 1:** Demographic characteristics of complete interviews by study arm.

		Bangladesh (n=1165), n (%) or n				Uganda (n=1248), n (%) or n		
		Control (n=393)	Promised incentive (n=413)	Lottery incentive (n=359)	Control (n=364)		Promised incentive (n=472)	Lottery incentive (n=412)
**Sex**								
	Male	353 (89.8)	369 (89.4)	320 (89.1)	276 (77.5)		357 (77.6)	315 (78.7)
	Female	39 (9.9)	44 (10.7)	37 (10.3)	80 (22.5)		103 (22.4)	85 (21.3)
	Other	1 (0.3)	0 (0.0)	2 (0.6)	N/A^a^		N/A	N/A
	Missing	0	0	0	8		12	12
**Age group (years)**								
	18-29	301 (76.6)	291 (70.5)	275 (76.6)	257 (72.2)		326 (71.0)	303 (75.6)
	30-49	75 (19.1)	109 (26.4)	71 (19.8)	91 (25.6)		124 (27.0)	92 (23.0)
	50-69	10 (2.5)	9 (2.2)	9 (2.5)	6 (1.7)		7 (1.5)	3 (0.8)
	70+	7 (1.8)	4 (1.0)	4 (1.1)	2 (0.6)		2 (0.4)	3 (0.8)
	Missing	0	0	0	8		13	11
**Education attempted**								
	None	29 (22.3)	34 (25.4)	28 (20.7)	65 (18.3)		63 (13.5)	59 (14.5)
	Primary	100 (76.9)	100 (74.6)	107 (79.3)	83 (23.4)		114 (24.4)	107 (26.3)
	Secondary	N/A	N/A	N/A	146 (41.1)		209 (44.8)	169 (41.5)
	Tertiary or higher	N/A	N/A	N/A	61 (17.2)		81 (17.3)	72 (17.7)
	Refused	1 (0.3)	0 (0.0)	0 (0.0)	0 (0.0)		0 (0.0)	0 (0.0)
	Missing	263	279	224	9		5	5
**Location**								
	Urban	225 (57.2)	222 (53.8)	180 (50.1)	178 (49.7)		250 (54.1)	227 (56.3)
	Rural	165 (42.0)	191 (46.2)	179 (49.9)	180 (50.3)		212 (45.9)	176 (43.7)
	Refused	3 (0.8)	0 (0.0)	0 (0.0)	0 (0.0)		0 (0.0)	0 (0.0)
	Missing	0	0	0	6		10	9
**Language**								
	Bangla	390 (99.2)	410 (99.3)	355 (98.9)	N/A		N/A	N/A
	English	3 (0.8)	3 (0.7)	4 (1.1)	56 (15.4)		68 (14.4)	66 (16.0)
	Luganda	N/A	N/A	N/A	213 (58.5)		260 (55.2)	248 (60.2)
	Luo	N/A	N/A	N/A	36 (9.9)		50 (10.6)	29 (7.0)
	Runyakitara	N/A	N/A	N/A	59 (16.2)		93 (19.8)	69 (16.8)
	Missing	0	0	0	0		1	0

^a^N/A: not applicable.

The sociodemographic characteristics of respondents with complete and partial interviews were similar in both countries, with the exception of a significant difference in age distribution in Bangladesh (*P*=.002); complete interviews had higher proportion of respondents aged 18 to 29 years old than partial interviews (Table S3 in Multimedia Appendix 1). The median time spent completing the interactive voice response survey was 15 minutes 8 seconds (IQR 14 minutes 8 seconds to 16 minutes 15 seconds) and 13 minutes 38 seconds (IQR 12 minutes 39 seconds to 14 minutes 45 seconds) in Bangladesh and Uganda, respectively. The direct costs of airtime, and incentives where applicable per complete interview were $3.89 and $3.16 in the control arm, $3.90 and $3.91 in the promised incentive arm, and $4.05 and $4.12 in the lottery incentive arm, in Bangladesh and Uganda, respectively (Table 2).

Cooperation and response rates were significantly higher in the promised incentive arm (cooperation: 413/1051, 39.3%; RR 1.38, 1.24-1.55, *P*<.001; response: 588/2222, 26.5%, RR 1.26, 95% CI 1.14-1.39, *P*<.001) and in the lottery arm (cooperation: 359/980, 36.6%; RR 1.28, 95% CI 1.15-1.45, *P*<.001; response: 544/2220, 24.5%; RR 1.17, 95% CI 1.06-1.29, *P*=.002) compared with those for the control arm (cooperation: 393/1383, 28.4%; response: 675/3216, 21.0%). In Uganda, the cooperation and response rates were higher than those in Bangladesh. Rates were significantly higher in the promised (cooperation: RR 1.47, 95% CI 1.33-1.62, *P*<.001; response: RR 1.27, 95% CI 1.16-1.39, *P*<.001) and lottery arms (cooperation: RR 1.34, 95% CI 1.21-1.48, *P*<.001; response: RR 1.17, 95% CI 1.06-1.29, *P*=.001) compared with those for the control arm. In both countries, cooperation and response rates were similar when using equations that did not include the estimated proportion of age-eligible participants in the unknown disposition code (Table S4 in Multimedia Appendix 1). In both countries, subgroup analyses showed that participant gender, age, education, and location did not modify the intervention’s effect on cooperation rate (Tables S5 and S6 in Multimedia Appendix 1).

**Figure 3:**
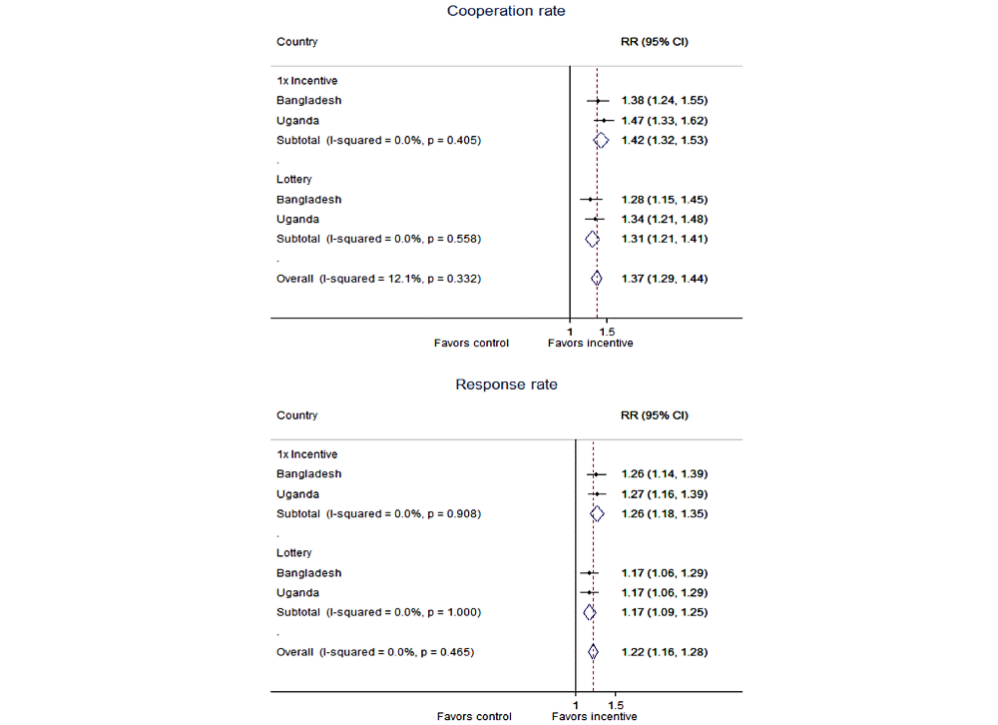
Pooled risk ratios for cooperation and response rate by study arm.

Pooling Bangladesh and Uganda participants showed that the promised incentive (pooled RR 1.42, 95% CI 1.32-1.53, *P<*0*.*001) and lottery incentive (pooled RR 1.31, 95% CI 1.21-1.41, *P<*0*.*001) significantly improved cooperation rate compared with no incentive (Figure 3). Similarly, response rates were significantly higher in the promised incentive (pooled RR 1.26, 95% CI 1.18-1.35, *P<*0*.*001) and lottery incentive (pooled RR 1.17, 95% CI 1.09-1.25, *P<*0*.*001*,* *I*^2^=0.0%) arm compared with that in the control arm. Overall, any incentive significantly improved cooperation rates by 37% (pooled RR 1.37, 95% CI 1.29-1.44, *P<*0*.*001) and response rates by 22% (pooled RR 1.22, 95% CI 1.18-1.28, *P*<*.*001), and these results were highly consistent (cooperation: *I*^2^=12.1%, *P*=.33; response: *I*^2^=0.0%, *P*=.47).

**Table 2:** Disposition codes and survey rates by study arm.

		Bangladesh			Uganda		
		Control	Promised incentive	Lottery incentive	Control	Promised incentive	Lottery incentive
**Complete interview, n**		393	413	359	364	472	412
**Partial interview, n**		282	175	185	201	131	133
**Refusal**							
	Refusal	402	296	265	145	96	94
	Breaks-off	306	167	171	181	89	116
**Unknown other, n**		2615	1671	1769	938	742	751
	Estimated unknown^a^	1833	1171	1240	854	675	684
**Ineligible, n**							
	Under age	587	465	402	99	59	79
	Call did not connect^b^	152,494	152,494	152,495	47,667	47,667	47,667
	Connected, but no language selection^b^	26,114	26,113	26,113	10,156	10,156	10,157
Average cost (US $) per complete interview^c^		3.89	3.90	4.05	3.16	3.91	4.12
**Contact rate**		43.00	47.30	44.10	51.10	53.90	52.50
	Risk ratio (95% CI)	Ref	1.10 (1.04-1.17)	1.03 (0.97-1.09)	Ref	1.05 (0.99-1.13)	1.03 (0.96-1.10)
	*P* value	Ref	.002	0.40	Ref	.11	.43
**Response rate**		21.00	26.50	24.50	32.40	41.20	37.90
	Risk ratio (95% CI)	Ref	1.26 (1.14-1.39)	1.17 (1.06-1.29)	Ref	1.27 (1.16-1.39)	1.17 (1.06-1.29)
	*P* value	Ref	<.001	.002	Ref	<.001	.001
**Refusal rate**		22.00	20.80	19.60	18.70	12.70	14.60
	Risk ratio (95% CI)	Ref	0.95 (0.85- 1.05)	0.89 (0.80- 0.99)	Ref	0.68 (0.57- 0.80)	0.78 (0.67- 0.92)
	*P* value	Ref	.30	.04	Ref	<.001	.002
**Cooperation rate**		28.40	39.30	36.60	40.90	59.90	54.60
	Risk ratio (95% CI)	Ref	1.38 (1.24-1.55)	1.28 (1.15-1.45)	Ref	1.47 (1.33-1.62)	1.34 (1.21-1.48)
	*P* value	Ref	<.001	<.001	Ref	<.001	<.001

^a^Estimated proportion of unknown cases that were age-eligible was 70.1% for Bangladesh and 91.0% for Uganda.

^b^Evenly distributed to each study arm due to randomization occurring after language selection.

^c^Only includes cost of the call based on time participants spent on the survey plus airtime incentive, as applicable.

## Discussion

In this study, the promised and lottery incentive arms had higher response and cooperation rates than control arms (ie, no incentive arm) in both Bangladesh and Uganda. Given that the pooled analysis showed that the incentives were highly consistent at increasing these survey rates in two culturally and geographically distinct low- and middle-income countries, the provision of airtime incentives may be a useful mechanism to increase interactive voice response survey participation in other low-resource settings.

There are very few studies [4,35] that have examined the use of airtime incentives in low- and middle-income countries. Our study adds significant knowledge to the growing body of literature on the impact of incentive amount on mobile phone survey in these countries. A previous study [4] similarly found that both promised and lottery airtime incentives significantly improved the completion rate of a random-digit dialing interactive voice response survey in Zimbabwe. In Mozambique, the lottery incentive, but not the promised incentive, increased completion rates [35].

A study [36] from Honduras also found that providing either US $1 or $5 of airtime significantly improved response rates compared with the no incentive arm. Another study [22] showed that providing an airtime incentive of at least 50 BDT in Bangladesh and 5000 UGX in Uganda improved survey participation compared to people without any incentive and also reduced the number of incomplete interviews.

Our interactive voice response survey data collection was quick and inexpensive. In Bangladesh, we collected 1165 complete interviews in 21 days at a cost of approximately US $4.00 per complete interview. In Uganda, 1248 complete interviews were collected in 18 days at a cost under US $4.00 per complete interview. Our findings are similar to those from a random-digit dialing interactive voice response survey in Ghana collected 9469 complete interviews in 27 days at a cost of US $4.95 per complete interview [37]. The average cost of a competed interview is much lower than the average cost of such a household survey, this indicates that the mobile phone survey could be cost-effective compared to household surveys. For instance, Lietz and colleagues [38] estimated the average cost of per completed interview of the Nouna Health and Demographic Survey in rural Burkina Faso as approximately US $25. Although the specific objectives of that survey were broader and required a longer amount of time than our survey, future studies should compare the average cost of conducting an interview in such mobile phone surveys. In Bangladesh, our use of an airtime incentive to motivate participants to complete the interview became cost-neutral compared to the control arm. The savings in cost was due to the decreased number of partial interviews and, therefore, fewer phone calls. We did not see a similar finding in Uganda where the promised (US $1.35) and lottery (US $28) incentive amounts were higher than those in Bangladesh (promised: US $0.60; lottery: US $6.00). The difference in promised incentive amount may also account for some differences in participation rate by country. Specifically, people may not initiate a survey if the promised incentive amount appears low, which would ultimately reduce participation. Future work could manipulate the odds of winning the lottery and its amount to ensure the incentive is cost-neutral or even cost-saving [39].

Our cooperation and response rates were calculated in a standardized manner using American Association for Public Opinion Research guidelines [31], which allows for comparison with other studies. In a nationally administered random-digit dialing interactive voice response survey, with persons ≥18 years in Ghana, in which no incentives were provided, contact (39%) and response (31%) rates were similar to those observed in our control arms for Bangladesh and Uganda [37]. However, we observed higher refusal and lower cooperation rates in Bangladesh (refusal: 22%; cooperation: 28%) and Uganda (refusal: 19%; cooperation: 41%) than what was observed in Ghana (refusal: 7%; cooperation: 59%). These differences may be explained by variations in the eligibility criteria, length of survey, and the classification of disposition codes for complete, break-offs, refusals, and partial interviews. For instance, L’Engle and colleagues [37] defined complete interviews as responding to all survey questions, while we defined complete as 4 out of 5 modules.

There are a range of ethical considerations in mobile phone survey [40]. Our survey started with an introduction that included the purpose of the study, the sponsoring agency, time commitment, and that the data would be kept confidential. Participants were offered an opportunity to consent to the survey by pressing a button on their mobile phone and were allowed to refuse to answer any question. Additional studies that evaluate alternative ways to consent participants are needed to maximize participant’s understanding of the study [41]. Additionally, there has been considerable discussion on the ethics of incentives and health research [42,43]. Our use of incentives was informed by in-country stakeholders, amounts used were less than a day’s working wage and were not paired with risky or unsafe behavior. Nonetheless, we believe important to acknowledge that efforts to optimize use of incentives, in general, should be informed not only by cost-effectiveness considerations. Incentives that insufficiently reflect response burden, or that, perhaps in rare cases, have the potential to unduly influence or induce participation, ought to be avoided.

We observed a higher proportion of male, young (ie, 18 to 29 years old), or urban residents compared to general population in both countries. This finding was similar to those of random-digit dialing interactive voice response surveys conducted in Afghanistan, Ethiopia, Ghana, Mozambique, Tanzania, and Zimbabwe [35,37,44]. Male gender, younger age, higher education, and urban residence have been found to be associated with mobile phone ownership in low- and middle-income countries, including East Africa [45] and Bangladesh [46]. This does raise concerns about the ability to generate nationally representative estimates (ie, generalizability of the findings). Advances in sampling and statistical methodology may be required for such estimates. Quota sampling could be used to ensure a more equal distribution of the sociodemographic characteristics [47]. Others have found that weighted estimates of noncommunicable disease indicators collected via mobile phone survey approximate household collected data [48].

This study has several strengths. First, the randomization was automated and embedded within the interactive voice response platform. This safeguarded against misallocation of participants to study arm which could bias response and cooperation rates. Second, we employed standardized protocols and questionnaires in both countries and used the same technology platform to deliver interactive voice response surveys to afford for cross-country comparisons. Lastly, our sampling frame consisted of all known mobile network operators in each country; thereby minimizing potential selection bias.

In addition to underrepresentation from some sociodemographic populations, this study has some limitations. First, there was a substantial number of phone calls in Bangladesh and Uganda where we were unable to determine the status of the phone numbers. Calling people randomly can also reduce response. We could not determine if the phone numbers we called were active or inactive numbers [49]. As randomization to study arm occurred after participants picked up the phone, we chose to designate these phone calls as nonworking numbers. This decision inflates our contact, response, and refusal rates, but has no effect on the cooperation rate. Second, although not an issue in Bangladesh where 99% of the respondents took the interactive voice response survey in Bangla, our survey was only available in 3 of the 6 major language groups in Uganda [50]. This might lead to some selection error due to unavailability of the preferred language and would have larger implications for nationally representative surveys [35]. We did not check the quality of collected data as that was not the main purpose of this study; future studies should investigate that.

We investigated the response, contact, and cooperation rates of 2 different incentive structures compared to providing no incentives in 2 geographically and linguistically, distinct countries. We observed that providing either type of incentive enhanced survey participation and minimized associated costs.

## Appendix

Multimedia Appendix 1 Supporting information.

Multimedia Appendix 2 CONSORT eHEALTH Checklist (V 1.6.1).

## Abbreviations

BDT: Bangladeshi Taka
RR: risk ratio
UGX: Ugandan Shilling

## Data Availability

All data files are available from the OpenICPSR repository database (accession number: ICPSR 107284).
