# Supplementary material for: Promised and Lottery Airtime Incentives to Improve Interactive Voice Response Survey Participation Among Adults in Bangladesh and Uganda: Randomized Controlled Trial
Source: J Med Internet Res. 2022 May 9;24(5):e36943. doi: 10.2196/36943 (PMC9127645; doi:10.2196/36943)
Supplement: Multimedia Appendix 1 [file jmir_v24i5e36943_app1.docx]

# SUPPORTING INFORMATION

Supporting Table 1: Survey Introduction in Bangladesh and Uganda

| Bangladesh | Uganda |
| --- | --- |
| Thank you. This interview will take about 20 minutes of your time. All information you provide will be kept confidential and private. To answer each question, I will ask you to press a certain number, for example, press 1 for YES, and 3 for NO or to answer a question with a number such as 12, tap 1 and 2. At any time, you can repeat the question by pressing the star button. If you are unwilling to answer any question, you can press 0. As a token of appreciation…ß | Hello, we are conducting a heath survey from Makerere University. This interview will take no more than 20 minutes of your time. Any information you share will be kept confidential and private. To answer each question, I will ask you to press a number - for example, 1 for YES, and 3 for NO, or to answer a question with a number - like 12 or 22. You can hear a question again at any time by pressing the STAR button - located to the left of the ZERO at the bottom of your phone. As a token of appreciation… |

**Supporting Table 2. Equations used to calculate survey rates**

|  | **Survey rate** | **Equation** |
| --- | --- | --- |
| **Primary analysis** | Contact Rate #2 | (I + P + R + O) / (I+ P + R + O + NC + *e*(UH+UO)) |
|  | Response Rate #4 | (I + P) / (I+ P + R + O + NC + *e*(UH+UO)) |
|  | Refusal Rate #2 | R / (I+ P + R + O + NC + *e*(UH+UO)) |
|  | Cooperation Rate #1 | I / (I + P +R + O) |
| **Secondary analysis** | Contact Rate #1 | (I + P + R + O) / (I + P + R + O + NC + UH + UO) |
|  | Response Rate #2 | (I + P) / (I + P + R + O + NC + UH + UO) |
|  | Refusal Rate #1 | R / (I + P + R + O + NC + UH + UO) |
|  | Cooperation Rate #2 | (I + P) / (I + P +R + O) |

Abbreviations: I, interview; P, partial interview; R, refusal/break-off; O, other; NC, non-contact; UH, unknown household; UO, unknown other; *e*, estimated proportion of unknown cases that were age-eligible

**Supporting table 3** Demographic characteristics of complete and partial interviews in Bangladesh and Uganda

|  | **Bangladesh** | | | **Uganda** | | |
| --- | --- | --- | --- | --- | --- | --- |
|  | **Complete Interviews**  **N = 1,165** | **Partial Interviews**  **N=642** | **p-value** | **Complete Interviews**  **N = 1,248** | **Partial Interviews**  **N=465** | **p-value** |
| **Sex (male)** | 1042 (89.2%) | 560 (87.2%) | 0.486 | 948 (78.0%) | 331 (73.9%) | 0.080 |
| **Age group (years)** |  |  |  |  |  |  |
| 18-29 | 867 (74.4%) | 440 (68.5%) | 0.002 | 886 (72.9%) | 328 (73.7%) | 0.060 |
| 30-49 | 255 (21.9%) | 164 (25.6%) |  | 307 (25.3%) | 99 (22.3%) |  |
| 50-69 | 28 (2.4%) | 15 (2.3%) |  | 16 (1.3%) | 12 (2.7%) |  |
| 70+ | 15 (1.3%) | 23 (3.6%) |  | 7 (0.6%) | 6 (1.4%) |  |
| Missing* | n=0 | n=0 |  | n=32 | n=20 |  |
| **Education attempted** |  |  |  |  |  |  |
| None | 91 (22.8%) | 68 (26.4%) | 0.185 | 187 (15.2%) | 80 (17.6%) | 0.650 |
| Primary | 307 (76.9%) | 187 (72.5%) |  | 304 (24.7%) | 107 (23.6%) |  |
| Secondary | NA | NA |  | 524 (42.6%) | 192 (42.3%) |  |
| Tertiary or higher | NA | NA |  | 214 (17.4%) | 74 (16.3%) |  |
| Refused | 1 (0.3%) | 3 (1.2%) |  | 0 (0.0%) | 1 (0.2%) |  |
| Missing*† | n=766 | n=384 |  | n=19 | n=11 |  |
| **Location** |  |  |  |  |  |  |
| Urban | 627 (53.8%) | 352 (54.8%) | 0.894 | 655 (53.6%) | 260 (57.8%) | 0.124 |
| Rural | 535 (45.9%) | 288 (44.9%) |  | 568 (46.4%) | 190 (42.2%) |  |
| Refused | 3 (0.3%) | 2 (0.3%) |  | 0 (0.0%) | 0 (0.0%) |  |
| Missing* | n=0 | n=0 |  | n=25 | n=15 |  |
| **Language** |  |  |  |  |  |  |
| Bangla | 1155 (99.1%) | 635 (98.9%) | 0.625 | NA | NA |  |
| English | 10 (0.9%) | 7 (1.1%) |  | 190 (15.2%) | 65 (14.0%) | 0.122 |
| Luganda | NA | NA |  | 721 (57.8%) | 297 (63.9%) |  |
| Luo | NA | NA |  | 115 (9.2%) | 32 (6.9%) |  |
| Runyakitara | NA | NA |  | 221 (17.7%) | 71 (15.3%) |  |
| Missing | n=0 | n=0 |  | n=1 | n=0 |  |

Data are n (%). Airtime incentives given after completion of survey *Missing values for Uganda are due to errors in platform that prevented storing data. †Missing values for Bangladesh are due to incorrect coding of IVR platform. UGX, Ugandan Shilling; NA, not applicable.

**Supporting Table 4**. Alternative survey rates by study arm

|  | **Bangladesh** | | | **Uganda** | | |
| --- | --- | --- | --- | --- | --- | --- |
|  | **Control** | **Promised incentive** | **Lottery Incentive** | **Control** | **Promised incentive** | **Lottery incentive** |
| **Contact Rate** (I + P + R + O) / (I + P + R + O + NC + UH + UO) | | | | | | |
| Equation #1 | 34.6% | 38.6% | 35.7% | 48.7% | 51.5% | 50.1% |
| Risk Ratio (95%CI) | *Ref.* | 1.12 (1.05–1.19) | 1.03 (0.96–1.10) | *Ref.* | 1.06 (0.99–1.13) | 1.03 (0.96–1.10) |
| p-value | *Ref.* | 0.0007 | 0.3705 | *Ref.* | 0.1068 | 0.4146 |
| **Response Rate** (I + P) / (I + P + R + O + NC + UH + UO) | | | | | | |
| Equation #2 | 16.9% | 21.6% | 19.8% | 30.9% | 39.4% | 36.2% |
| Risk Ratio (95%CI) | *Ref.* | 1.28 (1.16–1.41) | 1.17 (1.06–1.30) | *Ref.* | 1.28 (1.16–1.40) | 1.17 (1.06–1.29) |
| p-value | *Ref.* | <0.001 | 0.0023 | *Ref.* | <0.001 | 0.0012 |
| **Refusal Rate** R / (I + P + R + O + NC + UH + UO) | | | | | | |
| Equation #1 | 17.7% | 17.0% | 15.9% | 17.8% | 12.1% | 13.9% |
| Risk Ratio (95%CI) | *Ref.* | 0.96 (0.86– 1.07) | 0.90 (0.80– 1.00) | *Ref.* | 0.68 (0.57– 0.80) | 0.78 (0.67– 0.92) |
| p-value | *Ref.* | 0.4586 | 0.0474 | *Ref.* | <0.001 | 0.0025 |
| **Cooperation Rate** (I + P) / (I + P +R + O) | | | | | | |
| Equation #2 | 48.8% | 56.0% | 55.5% | 63.4% | 76.5% | 72.2% |
| Risk Ratio (95%CI) | *Ref.* | 1.15 (1.06–1.24) | 1.14 (1.05–1.23) | *Ref.* | 1.21 (1.13–1.29) | 1.14 (1.06–1.22) |
| p-value | *Ref.* | 0.0004 | 0.0012 | *Ref.* | <0.001 | 0.001 |

Abbreviations: I, complete interview; P, partial interview; R, refusal; O, other; NC, non-contact; UH, unknown household; UO, unknown other.

**Supporting Table 5**. Subgroup analyses of cooperation rates in Bangladesh

|  | Control | Promised incentive | Stratum-Specific RR | p-value* | Lottery Incentive | Stratum-Specific RR | p-value* |
| --- | --- | --- | --- | --- | --- | --- | --- |
| **Gender** |  |  |  |  |  |  |  |
| Male | 353/840 (42%) | 369/642 (57%) | 1.37 (1.23–1.52) | .51 | 320/609 (53%) | 1.25 (1.12–1.40) | .38 |
| Female | 39/88 (44%) | 44/81 (54%) | 1.23 (0.90–1.67) |  | 37/78 (47%) | 1.07 (0.77–1.49) |  |
| **Age** |  |  |  |  |  |  |  |
| < 25 years | 227/751 (30%) | 230/538 (43%) | 1.41 (1.22–1.64) | .73 | 204/496 (41%) | 1.36 (1.17–1.58) | .37 |
| > 25 years | 166/632 (26%) | 183/513 (36%) | 1.36 (1.14–1.62) |  | 155/484 (32%) | 1.22 (1.01–1.47) |  |
| **Education** |  |  |  |  |  |  |  |
| No school | 29/77 (38%) | 34/64 (53%) | 1.41 (0.97–2.04) | .93 | 28/57 (49%) | 1.30 (0.88–1.93) | .78 |
| Primary | 100/252 (40%) | 100/182 (55%) | 1.38 (1.13–1.69) |  | 107/194 (55%) | 1.39 (1.14–1.69) |  |
| **Location** |  |  |  |  |  |  |  |
| Urban | 225/537 (42%) | 222/379 (59%) | 1.40 (1.23–1.59) | .48 | 180/364 (49%) | 1.18 (1.02–1.36) | .38 |
| Rural | 165/366 (45%) | 191/325 (59%) | 1.30 (1.13–1.51) |  | 179/307 (58%) | 1.29 (1.12–1.50) |  |

Data are n/N (%) and RR (95%CI). Overall interaction terms were not significant for any of the sub-groups: gender, p=0.67; age, p=0.66; education, p=0.92; location, p=0.24. RR= Risk ratio. *p values obtained from an interaction term between intervention groups and demographic characteristic

**Supporting Table 6**. Subgroup analyses of cooperation rates in Uganda

|  | Control | Promised incentive | Stratum-Specific RR | p-value* | Lottery Incentive | Stratum-Specific RR | p-value* |
| --- | --- | --- | --- | --- | --- | --- | --- |
| **Gender** |  |  |  |  |  |  |  |
| Male | 276/549 (50%) | 357/510 (70%) | 1.39 (1.26–1.54) | .99 | 315/485 (65%) | 1.29 (1.16–1.44) | .80 |
| Female | 80/171 (47%) | 103/158 (65%) | 1.39 (1.15–1.70) |  | 85/145 (59%) | 1.25 (1.02–1.55) |  |
| **Age** |  |  |  |  |  |  |  |
| < 25 years | 198/489 (40%) | 243/418 (58%) | 1.44 (1.25–1.64) | .53 | 235/404 (58%) | 1.44 (1.25–1.65) | .098 |
| > 25 years | 158/378 (42%) | 216/338 (64%) | 1.53 (1.32–1.76) |  | 166/330 (50%) | 1.20 (1.03–1.41) |  |
| **Education** |  |  |  |  |  |  |  |
| < Primary | 148/278 (53%) | 177/263 (67%) | 1.26 (1.10–1.45) | .12 | 166/256 (65%) | 1.22 (1.06–1.40) | .24 |
| > Primary | 207/414 (50%) | 290/398 (73%) | 1.46 (1.30–1.63) |  | 241/354 (68%) | 1.36 (1.21–1.53) |  |
| **Location** |  |  |  |  |  |  |  |
| Urban | 178/397 (45%) | 250/374 (67%) | 1.49 (1.31–1.70) | .088 | 227/358 (63%) | 1.41 (1.24–1.62) | .091 |
| Rural | 180/318 (57%) | 212/293 (72%) | 1.28 (1.13–1.44) |  | 176/258 (68%) | 1.21 (1.06–1.37) |  |

Data are n/N (%) and RR (95%CI). Overall interaction terms were not significant for any of the sub-groups: gender, p=0.95; age, p=0.72; education, p=0.29; location, p=0.17. RR= Risk ratio. *p values obtained from an interaction term between intervention groups and demographic characteristic


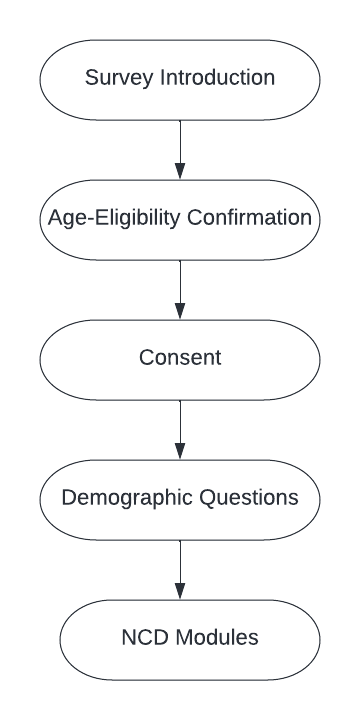


**Supporting Figure 1: Study procedure**
