# Supplementary material for: Promised and Lottery Airtime Incentives to Improve Interactive Voice Response Survey Participation Among Adults in Bangladesh and Uganda: Randomized Controlled Trial
Source: J Med Internet Res. 2022 May 9;24(5):e36943. doi: 10.2196/36943 (PMC9127645; doi:10.2196/36943)
Supplement: Multimedia Appendix 2 [file jmir_v24i5e36943_app2.pdf]

CONSORT-EHEALTH (V 1.6.1) - Submission/Publication Form

The CONSORT-EHEALTH checklist is intended for authors of randomized trials evaluating web-based and Internet-based applications/interventions, including mobile interventions, electronic games (incl multiplayer games), social media, certain telehealth applications, and other interactive and/or networked electronic applications. Some of the items (e.g. all subitems under item 5 - description of the intervention) may also be applicable for other study designs.

The goal of the CONSORT EHEALTH checklist and guideline is to be
a) a guide for reporting for authors of RCTs,
b) to form a basis for appraisal of an ehealth trial (in terms of validity)

CONSORT-EHEALTH items/subitems are MANDATORY reporting items for studies published in the Journal of Medical Internet Research and other journals / scientific societies endorsing the checklist.

Items numbered 1., 2., 3., 4a., 4b etc are original CONSORT or CONSORT-NPT (non-pharmacologic treatment) items.
Items with Roman numerals (i., ii, iii, iv etc.) are CONSORT-EHEALTH extensions/clarifications.

As the CONSORT-EHEALTH checklist is still considered in a formative stage, we would ask that you also RATE ON A SCALE OF 1-5 how important/useful you feel each item is FOR THE PURPOSE OF THE CHECKLIST and reporting guideline (optional).

Mandatory reporting items are marked with a red *.
In the textboxes, either copy & paste the relevant sections from your manuscript into this form - please include any quotes from your manuscript in QUOTATION MARKS,
or answer directly by providing additional information not in the manuscript, or elaborating on why the item was not relevant for this study.

YOUR ANSWERS WILL BE PUBLISHED AS A SUPPLEMENTARY FILE TO YOUR PUBLICATION IN JMIR AND ARE CONSIDERED PART OF YOUR PUBLICATION (IF ACCEPTED).
Please fill in these questions diligently. Information will not be copyedited, so please use proper spelling and grammar, use correct capitalization, and avoid abbreviations.

DO NOT FORGET TO SAVE AS PDF _AND_ CLICK THE SUBMIT BUTTON SO YOUR ANSWERS ARE IN OUR DATABASE !!!

Citation Suggestion (if you append the pdf as Appendix we suggest to cite this paper in the caption):
Eysenbach G, CONSORT-EHEALTH Group
CONSORT-EHEALTH: Improving and Standardizing Evaluation Reports of Web-based and Mobile Health Interventions

J Med Internet Res 2011;13(4):e126
URL: http://www.jmir.org/2011/4/e126/
doi: 10.2196/jmir.1923
PMID: 22209829

drkibriacomc13@gmail.com (not shared) Switch account

Draft saved

* Required

Your name *

First Last

Gulam Muhammed Al Kibria

Primary Affiliation (short), City, Country *

University of Toronto, Toronto, Canada

Baltimore, MD, USA

Your e-mail address *

abc@gmail.com

gkibria1@jhu.edu

Title of your manuscript *

Provide the (draft) title of your manuscript.

Airtime Incentives to Improve Interactive Voice Response Survey Participation among Adults in Bangladesh and Uganda: Randomized Controlled Trials to Test Impacts of Promised and Lottery Incentives

Name of your App/Software/Intervention *

If there is a short and a long/alternate name, write the short name first and add the long name in brackets.

Airtime incentives

Evaluated Version (if any)

e.g. "V1", "Release 2017-03-01", "Version 2.0.27913"

Your answer

Language(s) *

What language is the intervention/app in? If multiple languages are available, separate by comma (e.g. "English, French")

English

URL of your Intervention Website or App

e.g. a direct link to the mobile app on app in appstore (itunes, Google Play), or URL of the website. If the intervention is a DVD or hardware, you can also link to an Amazon page.

https://www.researchprotocols.org/2017/5/e81/

URL of an image/screenshot (optional)

Your answer

Accessibility *

Can an enduser access the intervention presently?

- ○ access is free and open
- ○ access only for special usergroups, not open
- ○ access is open to everyone, but requires payment/subscription/in-app purchases
- ◉ app/intervention no longer accessible
- ○ Other:

Primary Medical Indication/Disease/Condition *

e.g. "Stress", "Diabetes", or define the target group in brackets after the condition, e.g. "Autism (Parents of children with)", "Alzheimers (Informal Caregivers of)"

Mobile phone survey

Primary Outcomes measured in trial *

comma-separated list of primary outcomes reported in the trial

Cooperation rate and response rate

Secondary/other outcomes

Are there any other outcomes the intervention is expected to affect?

Contact rate and refusal rate

Recommended "Dose" *

What do the instructions for users say on how often the app should be used?

- ○ Approximately Daily
- ○ Approximately Weekly
- ○ Approximately Monthly
- ○ Approximately Yearly
- ○ "as needed"
- ● Other: Not applicable

Approx. Percentage of Users (starters) still using the app as recommended after 3 months *

- ○ unknown / not evaluated
- ● 0-10%
- ○ 11-20%
- ○ 21-30%
- ○ 31-40%
- ○ 41-50%
- ○ 51-60%
- ○ 61-70%
- ○ 71%-80%
- ○ 81-90%
- ○ 91-100%
- ○ Other:

Overall, was the app/intervention effective? *

- ○ yes: all primary outcomes were significantly better in intervention group vs control
- ○ partly: SOME primary outcomes were significantly better in intervention group vs control
- ○ no statistically significant difference between control and intervention
- ○ potentially harmful: control was significantly better than intervention in one or more outcomes
- ◉ inconclusive: more research is needed
- ○ Other:

Article Preparation Status/Stage *

At which stage in your article preparation are you currently (at the time you fill in this form)

- ○ not submitted yet - in early draft status
- ○ not submitted yet - in late draft status, just before submission
- ○ submitted to a journal but not reviewed yet
- ○ submitted to a journal and after receiving initial reviewer comments
- ◉ submitted to a journal and accepted, but not published yet
- ○ published
- ○ Other:

Journal *

If you already know where you will submit this paper (or if it is already submitted), please provide the journal name (if it is not JMIR, provide the journal name under "other")

- ○ not submitted yet / unclear where I will submit this
- ◉ Journal of Medical Internet Research (JMIR)
- ○ JMIR mHealth and UHealth
- ○ JMIR Serious Games
- ○ JMIR Mental Health
- ○ JMIR Public Health
- ○ JMIR Formative Research
- ○ Other JMIR sister journal
- ○ Other:

Is this a full powered effectiveness trial or a pilot/feasibility trial? *

- ○ Pilot/feasibility
- ◉ Fully powered

Manuscript tracking number *

If this is a JMIR submission, please provide the manuscript tracking number under "other" (The ms tracking number can be found in the submission acknowledgement email, or when you login as author in JMIR. If the paper is already published in JMIR, then the ms tracking number is the four-digit number at the end of the DOI, to be found at the bottom of each published article in JMIR)

- ○ no ms number (yet) / not (yet) submitted to / published in JMIR
- ◉ Other: 36943

TITLE AND ABSTRACT

1a) TITLE: Identification as a randomized trial in the title

1a) Does your paper address CONSORT item 1a? *

I.e does the title contain the phrase "Randomized Controlled Trial"? (if not, explain the reason under "other")

- (●) yes
- ( ) Other:

1a-i) Identify the mode of delivery in the title

Identify the mode of delivery. Preferably use “web-based” and/or “mobile” and/or “electronic game” in the title. Avoid ambiguous terms like “online”, “virtual”, “interactive”. Use “Internet-based” only if Intervention includes non-web-based Internet components (e.g. email), use “computer-based” or “electronic” only if offline products are used. Use “virtual” only in the context of “virtual reality” (3-D worlds). Use “online” only in the context of “online support groups”. Complement or substitute product names with broader terms for the class of products (such as “mobile” or “smart phone” instead of “iphone”), especially if the application runs on different platforms.

	1	2	3	4	5	
subitem not at all important	○	○	○	○	●	essential

Clear selection

Does your paper address subitem 1a-i? *

Copy and paste relevant sections from manuscript title (include quotes in quotation marks "like this" to indicate direct quotes from your manuscript), or elaborate on this item by providing additional information not in the ms, or briefly explain why the item is not applicable/relevant for your study

"Airtime Incentives to Improve Interactive Voice Response Survey Participation among Adults in Bangladesh and Uganda"

1a-ii) Non-web-based components or important co-interventions in title

Mention non-web-based components or important co-interventions in title, if any (e.g., “with telephone support”).

	1	2	3	4	5	
subitem not at all important	(•)	( )	( )	( )	( )	essential

Clear selection

Does your paper address subitem 1a-ii?

Copy and paste relevant sections from manuscript title (include quotes in quotation marks "like this" to indicate direct quotes from your manuscript), or elaborate on this item by providing additional information not in the ms, or briefly explain why the item is not applicable/relevant for your study

Your answer

1a-iii) Primary condition or target group in the title

Mention primary condition or target group in the title, if any (e.g., “for children with Type I Diabetes”)
Example: A Web-based and Mobile Intervention with Telephone Support for Children with Type I Diabetes: Randomized Controlled Trial

	1	2	3	4	5	
subitem not at all important	( )	( )	( )	( )	(•)	essential

Clear selection

Does your paper address subitem 1a-iii? *

Copy and paste relevant sections from manuscript title (include quotes in quotation marks "like this" to indicate direct quotes from your manuscript), or elaborate on this item by providing additional information not in the ms, or briefly explain why the item is not applicable/relevant for your study

"among Adults in Bangladesh and Uganda"

1b) ABSTRACT: Structured summary of trial design, methods, results, and conclusions

NPT extension: Description of experimental treatment, comparator, care providers, centers, and blinding status.

1b-i) Key features/functionalities/components of the intervention and comparator in the METHODS section of the ABSTRACT

Mention key features/functionalities/components of the intervention and comparator in the abstract. If possible, also mention theories and principles used for designing the site. Keep in mind the needs of systematic reviewers and indexers by including important synonyms. (Note: Only report in the abstract what the main paper is reporting. If this information is missing from the main body of text, consider adding it)

	1	2	3	4	5	
subitem not at all important	○	○	○	○	◉	essential

Clear selection

Does your paper address subitem 1b-i? *

Copy and paste relevant sections from the manuscript abstract (include quotes in quotation marks "like this" to indicate direct quotes from your manuscript), or elaborate on this item by providing additional information not in the ms, or briefly explain why the item is not applicable/relevant for your study

"The open-label randomized controlled trial had three arms: 1) control arm with no incentive; 2) promised airtime incentive: 50 Bangladeshi Taka (BDT; $0.6) or 5000 Ugandan Shilling (UGX; $1.35); or 3) lottery incentive (500 BDT and 100,000 UGX) where the odds of winning the lottery were 1:20."

1b-ii) Level of human involvement in the METHODS section of the ABSTRACT

Clarify the level of human involvement in the abstract, e.g., use phrases like “fully automated” vs. “therapist/nurse/care provider/physician-assisted” (mention number and expertise of providers involved, if any). (Note: Only report in the abstract what the main paper is reporting. If this information is missing from the main body of text, consider adding it)

	1	2	3	4	5	
subitem not at all important	○	○	○	○	◉	essential

Clear selection

Does your paper address subitem 1b-ii?

Copy and paste relevant sections from the manuscript abstract (include quotes in quotation marks "like this" to indicate direct quotes from your manuscript), or elaborate on this item by providing additional information not in the ms, or briefly explain why the item is not applicable/relevant for your study

"Fully automated random digit dialing was used to sample eligible participants aged ≥18 years."

1b-iii) Open vs. closed, web-based (self-assessment) vs. face-to-face assessments in the METHODS section of the ABSTRACT

Mention how participants were recruited (online vs. offline), e.g., from an open access website or from a clinic or a closed online user group (closed usergroup trial), and clarify if this was a purely web-based trial, or there were face-to-face components (as part of the intervention or for assessment). Clearly say if outcomes were self-assessed through questionnaires (as common in web-based trials). Note: In traditional offline trials, an open trial (open-label trial) is a type of clinical trial in which both the researchers and participants know which treatment is being administered. To avoid confusion, use “blinded” or “unblinded” to indicated the level of blinding instead of “open”, as “open” in web-based trials usually refers to “open access” (i.e. participants can self-enrol). (Note: Only report in the abstract what the main paper is reporting. If this information is missing from the main body of text, consider adding it)

	1	2	3	4	5	
subitem not at all important	○	○	○	○	◉	essential

Clear selection

Does your paper address subitem 1b-iii?

Copy and paste relevant sections from the manuscript abstract (include quotes in quotation marks "like this" to indicate direct quotes from your manuscript), or elaborate on this item by providing additional information not in the ms, or briefly explain why the item is not applicable/relevant for your study

"random digit dialing was used to sample eligible participants aged ≥18 years."

1b-iv) RESULTS section in abstract must contain use data

Report number of participants enrolled/assessed in each group, the use/uptake of the intervention (e.g., attrition/adherence metrics, use over time, number of logins etc.), in addition to primary/secondary outcomes. (Note: Only report in the abstract what the main paper is reporting. If this information is missing from the main body of text, consider adding it)

	1	2	3	4	5	
subitem not at all important	○	◉	○	○	○	essential

Clear selection

Does your paper address subitem 1b-iv?

Copy and paste relevant sections from the manuscript abstract (include quotes in quotation marks "like this" to indicate direct quotes from your manuscript), or elaborate on this item by providing additional information not in the ms, or briefly explain why the item is not applicable/relevant for your study

"Between June 14 and July 14, 2017, and March 26 and April 22, 2017, 546,746 (1,165) and 178,572 (1,248) phone calls (complete interviews) were made, respectively, in Bangladesh and Uganda."

1b-v) CONCLUSIONS/DISCUSSION in abstract for negative trials

Conclusions/Discussions in abstract for negative trials: Discuss the primary outcome - if the trial is negative (primary outcome not changed), and the intervention was not used, discuss whether negative results are attributable to lack of uptake and discuss reasons. (Note: Only report in the abstract what the main paper is reporting. If this information is missing from the main body of text, consider adding it)

	1	2	3	4	5	
subitem not at all important	(•)	( )	( )	( )	( )	essential

Clear selection

Does your paper address subitem 1b-v?

Copy and paste relevant sections from the manuscript abstract (include quotes in quotation marks "like this" to indicate direct quotes from your manuscript), or elaborate on this item by providing additional information not in the ms, or briefly explain why the item is not applicable/relevant for your study

Your answer

INTRODUCTION

2a) In INTRODUCTION: Scientific background and explanation of rationale

2a-i) Problem and the type of system/solution

Describe the problem and the type of system/solution that is object of the study: intended as stand-alone intervention vs. incorporated in broader health care program? Intended for a particular patient population? Goals of the intervention, e.g., being more cost-effective to other interventions, replace or complement other solutions? (Note: Details about the intervention are provided in “Methods” under 5)

	1	2	3	4	5	
subitem not at all important	( )	( )	( )	( )	( )	essential

Does your paper address subitem 2a-i? *

Copy and paste relevant sections from the manuscript (include quotes in quotation marks "like this" to indicate direct quotes from your manuscript), or elaborate on this item by providing additional information not in the ms, or briefly explain why the item is not applicable/relevant for your study

Yes, "There are several options for delivering MPS; these include short message service (SMS), interviewing respondents from call centers by a human operator, and interactive voice response (IVR) [14]. IVR is an MPS method where respondents use their mobile phone keypad to select answers from pre-specified options (e.g., "Press 1 if you are male, press 2 if you are female"). An incentive, often delivered as cash, coupon, voucher, or airtime balance for MPS, have been shown to increase survey participation [15]. It could also be considered as a compensation for the time spent by participants. Incentives could reduce the amount of time required for data collection by recruiting optimum number of participants in a shorter time period. In HICs, where there is a larger body of evidence, providing an incentive prior to the survey (i.e., prepaid) typically produces better survey response than promised or lottery incentives across a range of different survey types [17,19,20]. However, the overall findings were mixed and some studies show that providing incentives does not improve participation [21,22]. Past studies from LMICs also showed similar mixed results. Studies have also shown that delivering incentives to everyone can increase participation and reduce cost than using lottery alone [16,18]. In addition to participation, there has been limited number of studies examining the impact of different incentive amount on the overall survey cost, investigating these would be helpful to understand the feasibility of MPS for future data collection [23,24]. "

2a-ii) Scientific background, rationale: What is known about the (type of) system

Scientific background, rationale: What is known about the (type of) system that is the object of the study (be sure to discuss the use of similar systems for other conditions/diagnoses, if appropiate), motivation for the study, i.e. what are the reasons for and what is the context for this specific study, from which stakeholder viewpoint is the study performed, potential impact of findings [2]. Briefly justify the choice of the comparator.

	1	2	3	4	5	
subitem not at all important	○	○	○	○	◉	essential

Clear selection

Does your paper address subitem 2a-ii? *

Copy and paste relevant sections from the manuscript (include quotes in quotation marks "like this" to indicate direct quotes from your manuscript), or elaborate on this item by providing additional information not in the ms, or briefly explain why the item is not applicable/relevant for your study

"There are several options for delivering MPS; these include short message service (SMS), interviewing respondents from call centers by a human operator, and interactive voice response (IVR) [14]. IVR is an MPS method where respondents use their mobile phone keypad to select answers from pre-specified options (e.g., "Press 1 if you are male, press 2 if you are female"). An incentive, often delivered as cash, coupon, voucher, or airtime balance for MPS, have been shown to increase survey participation [15]. It could also be considered as a compensation for the time spent by participants. Incentives could reduce the amount of time required for data collection by recruiting optimum number of participants in a shorter time period. In HICs, where there is a larger body of evidence, providing an incentive prior to the survey (i.e., prepaid) typically produces better survey response than promised or lottery incentives across a range of different survey types [17,19,20]. However, the overall findings were mixed and some studies show that providing incentives does not improve participation [21,22]. Past studies from LMICs also showed similar mixed results. Studies have also shown that delivering incentives to everyone can increase participation and reduce cost than using lottery alone [16,18]. In addition to participation, there has been limited number of studies examining the impact of different incentive amount on the overall survey cost, investigating these would be helpful to understand the feasibility of MPS for future data collection [23,24]. "

2b) In INTRODUCTION: Specific objectives or hypotheses

Does your paper address CONSORT subitem 2b? *

Copy and paste relevant sections from the manuscript (include quotes in quotation marks "like this" to indicate direct quotes from your manuscript), or elaborate on this item by providing additional information not in the ms, or briefly explain why the item is not applicable/relevant for your study

Yes, "This study aims to fill these existing gaps in the literature by assessing the effect of different airtime incentive approaches on cooperation, response, contact, and refusal rates of an IVR survey for NCD behavioral risk factors. "

METHODS

3a) Description of trial design (such as parallel, factorial) including allocation ratio

Does your paper address CONSORT subitem 3a? *

Copy and paste relevant sections from the manuscript (include quotes in quotation marks "like this" to indicate direct quotes from your manuscript), or elaborate on this item by providing additional information not in the ms, or briefly explain why the item is not applicable/relevant for your study

Yes, "We conducted a randomized controlled trial in Bangladesh and Uganda. With an area of about 148,000 km2, the estimated population of Bangladesh is 160 million. The total area of Uganda is about 241,000 km2, and the estimated population is about 40 million [25]. In 2017, mobile phone subscription rates were 83 and 55 subscribers per 100 people, in Bangladesh and Uganda, respectively [9].
In this trial, incentives were delivered as airtime (i.e., mobile phone balance). Participants were randomized to one of three study arms: 1) no incentive (control group); 2) a promised airtime incentive of 50 Bangladeshi Taka (BDT; $0.6 USD) or 5000 Ugandan Shilling (UGX; $1.35 USD) for completing the IVR survey; or 3) lottery incentive (500 BDT and 100,000 UGX) where the odds of winning the lottery were 1:20. The conduct, analysis, and reporting of results were done in accordance with the Consolidated Standards of Reporting Trials guidelines [26]."

3b) Important changes to methods after trial commencement (such as eligibility criteria), with reasons

Does your paper address CONSORT subitem 3b? *

Copy and paste relevant sections from the manuscript (include quotes in quotation marks "like this" to indicate direct quotes from your manuscript), or elaborate on this item by providing additional information not in the ms, or briefly explain why the item is not applicable/relevant for your study

Not applicable to this study

3b-i) Bug fixes, Downtimes, Content Changes

Bug fixes, Downtimes, Content Changes: ehealth systems are often dynamic systems. A description of changes to methods therefore also includes important changes made on the intervention or comparator during the trial (e.g., major bug fixes or changes in the functionality or content) (5-iii) and other “unexpected events” that may have influenced study design such as staff changes, system failures/downtimes, etc. [2].

	1	2	3	4	5	
subitem not at all important	(•)	( )	( )	( )	( )	essential

Clear selection

Does your paper address subitem 3b-i?

Copy and paste relevant sections from the manuscript (include quotes in quotation marks "like this" to indicate direct quotes from your manuscript), or elaborate on this item by providing additional information not in the ms, or briefly explain why the item is not applicable/relevant for your study

Not applicable to this study

4a) Eligibility criteria for participants

Does your paper address CONSORT subitem 4a? *

Copy and paste relevant sections from the manuscript (include quotes in quotation marks "like this" to indicate direct quotes from your manuscript), or elaborate on this item by providing additional information not in the ms, or briefly explain why the item is not applicable/relevant for your study

Yes, "Respondents who self-reported being aged 18 years or older were eligible for the trial. "

4a-i) Computer / Internet literacy

Computer / Internet literacy is often an implicit "de facto" eligibility criterion - this should be explicitly clarified.

	1	2	3	4	5	
subitem not at all important	(●)	( )	( )	( )	( )	essential

Clear selection

Does your paper address subitem 4a-i?

Copy and paste relevant sections from the manuscript (include quotes in quotation marks "like this" to indicate direct quotes from your manuscript), or elaborate on this item by providing additional information not in the ms, or briefly explain why the item is not applicable/relevant for your study

Not applicable to this study

4a-ii) Open vs. closed, web-based vs. face-to-face assessments:

Open vs. closed, web-based vs. face-to-face assessments: Mention how participants were recruited (online vs. offline), e.g., from an open access website or from a clinic, and clarify if this was a purely web-based trial, or there were face-to-face components (as part of the intervention or for assessment), i.e., to what degree got the study team to know the participant. In online-only trials, clarify if participants were quasi-anonymous and whether having multiple identities was possible or whether technical or logistical measures (e.g., cookies, email confirmation, phone calls) were used to detect/prevent these.

	1	2	3	4	5	
subitem not at all important	( )	( )	( )	(●)	( )	essential

Clear selection

Does your paper address subitem 4a-ii? *

Copy and paste relevant sections from the manuscript (include quotes in quotation marks "like this" to indicate direct quotes from your manuscript), or elaborate on this item by providing additional information not in the ms, or briefly explain why the item is not applicable/relevant for your study

"The automated randomization process was done within the IVR platform to cover all mobile phone networks in both countries. Participants were randomized after selecting the survey language, but prior to consent (Supporting Figure 1). Due to the nature of the study design, participants were informed about the incentive during the survey introduction and therefore not blinded to study arm allocation. Statisticians involved in data cleaning and analysis were blinded to participants' allocation group. "

4a-iii) Information giving during recruitment

Information given during recruitment. Specify how participants were briefed for recruitment and in the informed consent procedures (e.g., publish the informed consent documentation as appendix, see also item X26), as this information may have an effect on user self-selection, user expectation and may also bias results.

	1	2	3	4	5	
subitem not at all important	○	○	○	○	●	essential

Clear selection

Does your paper address subitem 4a-iii?

Copy and paste relevant sections from the manuscript (include quotes in quotation marks "like this" to indicate direct quotes from your manuscript), or elaborate on this item by providing additional information not in the ms, or briefly explain why the item is not applicable/relevant for your study

Added in the appendix, "The overall procedures were similar in both countries. IVR surveys were sent only once to each phone number and were delivered between 8:00 AM and 8:00 PM local time. Respondents who picked up the phone were instructed to select a language from a list of languages; Bangla or English in Bangladesh and Luganda, Luo, Runyakitara, or English in Uganda. Then, participants listened to a survey introduction where the survey objectives and requirements for the incentive (i.e., survey completion), as applicable, were described (Supporting Table 1 and Supporting Figure 1). Participants were told that they would not incur any expenses by answering the survey. Then, age-eligibility was confirmed (i.e., "Are you 18 years or older? If Yes, Press 1. If No, Press 3"). Age-eligible participants were provided the consent disclosure statement and requested to authorize their participation by pressing the 1 button on the mobile phone. Next, they answered demographic and NCD questions. Only those who completed the survey received the incentive. Participants were instructed to press the 'star' key to repeat any questions."

4b) Settings and locations where the data were collected

Does your paper address CONSORT subitem 4b? *

Copy and paste relevant sections from the manuscript (include quotes in quotation marks "like this" to indicate direct quotes from your manuscript), or elaborate on this item by providing additional information not in the ms, or briefly explain why the item is not applicable/relevant for your study

Yes, "We conducted a randomized controlled trial in Bangladesh and Uganda. With an area of about 148,000 km2, the estimated population of Bangladesh is 160 million. The total area of Uganda is about 241,000 km2, and the estimated population is about 40 million [25]. In 2017, mobile phone subscription rates were 83 and 55 subscribers per 100 people, in Bangladesh and Uganda, respectively [9]. "

"The automated randomization process was done within the IVR platform to cover all mobile phone networks in both countries."

4b-i) Report if outcomes were (self-)assessed through online questionnaires

Clearly report if outcomes were (self-)assessed through online questionnaires (as common in web-based trials) or otherwise.

	1	2	3	4	5	
subitem not at all important	(●)	( )	( )	( )	( )	essential

Clear selection

Does your paper address subitem 4b-i? *

Copy and paste relevant sections from the manuscript (include quotes in quotation marks "like this" to indicate direct quotes from your manuscript), or elaborate on this item by providing additional information not in the ms, or briefly explain why the item is not applicable/relevant for your study

Not applicable to this study

4b-ii) Report how institutional affiliations are displayed

Report how institutional affiliations are displayed to potential participants [on ehealth media], as affiliations with prestigious hospitals or universities may affect volunteer rates, use, and reactions with regards to an intervention.(Not a required item – describe only if this may bias results)

	1	2	3	4	5	
subitem not at all important	( )	( )	( )	( )	(●)	essential

Clear selection

Does your paper address subitem 4b-ii?

Copy and paste relevant sections from the manuscript (include quotes in quotation marks "like this" to indicate direct quotes from your manuscript), or elaborate on this item by providing additional information not in the ms, or briefly explain why the item is not applicable/relevant for your study

We added that in the appendix

5) The interventions for each group with sufficient details to allow replication, including how and when they were actually administered

5-i) Mention names, credential, affiliations of the developers, sponsors, and owners

Mention names, credential, affiliations of the developers, sponsors, and owners [6] (if authors/evaluators are owners or developer of the software, this needs to be declared in a “Conflict of interest” section or mentioned elsewhere in the manuscript).

	1	2	3	4	5	
subitem not at all important	◉	○	○	○	○	essential

Clear selection

Does your paper address subitem 5-i?

Copy and paste relevant sections from the manuscript (include quotes in quotation marks "like this" to indicate direct quotes from your manuscript), or elaborate on this item by providing additional information not in the ms, or briefly explain why the item is not applicable/relevant for your study

Not applicable to this study

5-ii) Describe the history/development process

Describe the history/development process of the application and previous formative evaluations (e.g., focus groups, usability testing), as these will have an impact on adoption/use rates and help with interpreting results.

	1	2	3	4	5	
subitem not at all important	○	○	○	○	◉	essential

Clear selection

Does your paper address subitem 5-ii?

Copy and paste relevant sections from the manuscript (include quotes in quotation marks "like this" to indicate direct quotes from your manuscript), or elaborate on this item by providing additional information not in the ms, or briefly explain why the item is not applicable/relevant for your study

"The questionnaire was adopted from standardized surveys including the WHO STEPwise Approach to NCD Risk Factor Surveillance (STEPS) and STEPwise Surveillance and Tobacco Questions for Surveys [28]. Initial cognitive testing and user-groups were conducted with people who identified themselves from an LMIC at Johns Hopkins University [29]. A series of key informant interviews and focus group discussions were conducted in each country to assess the comprehensibility and accuracy of the translated questionnaire and to improve the usability of the IVR platform."

5-iii) Revisions and updating

Revisions and updating. Clearly mention the date and/or version number of the application/intervention (and comparator, if applicable) evaluated, or describe whether the intervention underwent major changes during the evaluation process, or whether the development and/or content was “frozen” during the trial. Describe dynamic components such as news feeds or changing content which may have an impact on the replicability of the intervention (for unexpected events see item 3b).

	1	2	3	4	5	
subitem not at all important	(●)	( )	( )	( )	( )	essential

Clear selection

Does your paper address subitem 5-iii?

Copy and paste relevant sections from the manuscript (include quotes in quotation marks "like this" to indicate direct quotes from your manuscript), or elaborate on this item by providing additional information not in the ms, or briefly explain why the item is not applicable/relevant for your study

not applicable to this study

5-iv) Quality assurance methods

Provide information on quality assurance methods to ensure accuracy and quality of information provided [1], if applicable.

	1	2	3	4	5	
subitem not at all important	○	○	○	●	○	essential

Clear selection

Does your paper address subitem 5-iv?

Copy and paste relevant sections from the manuscript (include quotes in quotation marks "like this" to indicate direct quotes from your manuscript), or elaborate on this item by providing additional information not in the ms, or briefly explain why the item is not applicable/relevant for your study

"The questionnaire was adopted from standardized surveys including the WHO STEPwise Approach to NCD Risk Factor Surveillance (STEPS) and STEPwise Surveillance and Tobacco Questions for Surveys [28]. Initial cognitive testing and user-groups were conducted with people who identified themselves from an LMIC at Johns Hopkins University [29]. A series of key informant interviews and focus group discussions were conducted in each country to assess the comprehensibility and accuracy of the translated questionnaire and to improve the usability of the IVR platform."

5-v) Ensure replicability by publishing the source code, and/or providing screenshots/screen-capture video, and/or providing flowcharts of the algorithms used

Ensure replicability by publishing the source code, and/or providing screenshots/screen-capture video, and/or providing flowcharts of the algorithms used. Replicability (i.e., other researchers should in principle be able to replicate the study) is a hallmark of scientific reporting.

	1	2	3	4	5	
subitem not at all important	●	○	○	○	○	essential

Clear selection

Does your paper address subitem 5-v?

Copy and paste relevant sections from the manuscript (include quotes in quotation marks "like this" to indicate direct quotes from your manuscript), or elaborate on this item by providing additional information not in the ms, or briefly explain why the item is not applicable/relevant for your study

Not applicable to this study

5-vi) Digital preservation

Digital preservation: Provide the URL of the application, but as the intervention is likely to change or disappear over the course of the years; also make sure the intervention is archived (Internet Archive, webcitation.org, and/or publishing the source code or screenshots/videos alongside the article). As pages behind login screens cannot be archived, consider creating demo pages which are accessible without login.

	1	2	3	4	5	
subitem not at all important	◉	○	○	○	○	essential

Clear selection

Does your paper address subitem 5-vi?

Copy and paste relevant sections from the manuscript (include quotes in quotation marks "like this" to indicate direct quotes from your manuscript), or elaborate on this item by providing additional information not in the ms, or briefly explain why the item is not applicable/relevant for your study

Not applicable to this study

5-vii) Access

Access: Describe how participants accessed the application, in what setting/context, if they had to pay (or were paid) or not, whether they had to be a member of specific group. If known, describe how participants obtained "access to the platform and Internet" [1]. To ensure access for editors/reviewers/readers, consider to provide a "backdoor" login account or demo mode for reviewers/readers to explore the application (also important for archiving purposes, see vi).

	1	2	3	4	5	
subitem not at all important	○	○	◉	○	○	essential

Clear selection

Does your paper address subitem 5-vii? *

Copy and paste relevant sections from the manuscript (include quotes in quotation marks "like this" to indicate direct quotes from your manuscript), or elaborate on this item by providing additional information not in the ms, or briefly explain why the item is not applicable/relevant for your study

"The overall procedures were similar in both countries. IVR surveys were sent only once to each phone number and were delivered between 8:00 AM and 8:00 PM local time. Respondents who picked up the phone were instructed to select a language from a list of languages; Bangla or English in Bangladesh and Luganda, Luo, Runyakitara, or English in Uganda. Then, participants listened to a survey introduction where the survey objectives and requirements for the incentive (i.e., survey completion), as applicable, were described (Supporting Table 1 and Supporting Figure 1). Participants were told that they would not incur any expenses by answering the survey. Then, age-eligibility was confirmed (i.e., "Are you 18 years or older? If Yes, Press 1. If No, Press 3"). Age-eligible participants were provided the consent disclosure statement and requested to authorize their participation by pressing the 1 button on the mobile phone. Next, they answered demographic and NCD questions. Only those who completed the survey received the incentive. Participants were instructed to press the 'star' key to repeat any questions. "

5-viii) Mode of delivery, features/functionalities/components of the intervention and comparator, and the theoretical framework

Describe mode of delivery, features/functionalities/components of the intervention and comparator, and the theoretical framework [6] used to design them (instructional strategy [1], behaviour change techniques, persuasive features, etc., see e.g., [7, 8] for terminology). This includes an in-depth description of the content (including where it is coming from and who developed it) [1],” whether [and how] it is tailored to individual circumstances and allows users to track their progress and receive feedback” [6]. This also includes a description of communication delivery channels and – if computer-mediated communication is a component – whether communication was synchronous or asynchronous [6]. It also includes information on presentation strategies [1], including page design principles, average amount of text on pages, presence of hyperlinks to other resources, etc. [1].

	1	2	3	4	5	
subitem not at all important	○	○	○	○	◉	essential

Clear selection

Does your paper address subitem 5-viii? *

Copy and paste relevant sections from the manuscript (include quotes in quotation marks "like this" to indicate direct quotes from your manuscript), or elaborate on this item by providing additional information not in the ms, or briefly explain why the item is not applicable/relevant for your study

"The automated randomization process was done within the IVR platform to cover all mobile phone networks in both countries. "

5-ix) Describe use parameters

Describe use parameters (e.g., intended “doses” and optimal timing for use). Clarify what instructions or recommendations were given to the user, e.g., regarding timing, frequency, heaviness of use, if any, or was the intervention used ad libitum.

	1	2	3	4	5	
subitem not at all important	◉	○	○	○	○	essential

Clear selection

Does your paper address subitem 5-ix?

Copy and paste relevant sections from the manuscript (include quotes in quotation marks "like this" to indicate direct quotes from your manuscript), or elaborate on this item by providing additional information not in the ms, or briefly explain why the item is not applicable/relevant for your study

Not applicable to this study

5-x) Clarify the level of human involvement

Clarify the level of human involvement (care providers or health professionals, also technical assistance) in the e-intervention or as co-intervention (detail number and expertise of professionals involved, if any, as well as “type of assistance offered, the timing and frequency of the support, how it is initiated, and the medium by which the assistance is delivered”. It may be necessary to distinguish between the level of human involvement required for the trial, and the level of human involvement required for a routine application outside of a RCT setting (discuss under item 21 – generalizability).

	1	2	3	4	5	
subitem not at all important	(•)	( )	( )	( )	( )	essential

Clear selection

Does your paper address subitem 5-x?

Copy and paste relevant sections from the manuscript (include quotes in quotation marks "like this" to indicate direct quotes from your manuscript), or elaborate on this item by providing additional information not in the ms, or briefly explain why the item is not applicable/relevant for your study

Not applicable to this study

5-xi) Report any prompts/reminders used

Report any prompts/reminders used: Clarify if there were prompts (letters, emails, phone calls, SMS) to use the application, what triggered them, frequency etc. It may be necessary to distinguish between the level of prompts/reminders required for the trial, and the level of prompts/reminders for a routine application outside of a RCT setting (discuss under item 21 – generalizability).

	1	2	3	4	5	
subitem not at all important	(●)	○	○	○	○	essential

Clear selection

Does your paper address subitem 5-xi? *

Copy and paste relevant sections from the manuscript (include quotes in quotation marks "like this" to indicate direct quotes from your manuscript), or elaborate on this item by providing additional information not in the ms, or briefly explain why the item is not applicable/relevant for your study

Not applicable to this study

5-xii) Describe any co-interventions (incl. training/support)

Describe any co-interventions (incl. training/support): Clearly state any interventions that are provided in addition to the targeted eHealth intervention, as ehealth intervention may not be designed as stand-alone intervention. This includes training sessions and support [1]. It may be necessary to distinguish between the level of training required for the trial, and the level of training for a routine application outside of a RCT setting (discuss under item 21 – generalizability.

	1	2	3	4	5	
subitem not at all important	(●)	○	○	○	○	essential

Clear selection

Does your paper address subitem 5-xii? *

Copy and paste relevant sections from the manuscript (include quotes in quotation marks "like this" to indicate direct quotes from your manuscript), or elaborate on this item by providing additional information not in the ms, or briefly explain why the item is not applicable/relevant for your study

Not applicable to this study

6a) Completely defined pre-specified primary and secondary outcome measures, including how and when they were assessed

Does your paper address CONSORT subitem 6a? *

Copy and paste relevant sections from the manuscript (include quotes in quotation marks "like this" to indicate direct quotes from your manuscript), or elaborate on this item by providing additional information not in the ms, or briefly explain why the item is not applicable/relevant for your study

"The primary outcomes of this trial were response rates #4 and cooperation rates #1 as defined by the American Association for Public Opinion Research (AAPOR; Supporting Table 2) [31]. Response rate includes partial and complete surveys in the numerator. Cooperation rate is the proportion of complete interviews from all eligible respondents, it does not include people who immediately hang up or do not answer the age question in the denominator. Secondary outcomes included contact rate #2 and refusal rate #2. The cooperation rate was the number of complete interviews divided by the sum of complete, partial, and non-interviews. Complete interviews (I) were defined as respondents who answered at least 4 of the 5 NCD modules. Partial interviews (PI) were defined as respondents who answered between 1 and 3 NCD modules. Non-interviews included refusals (i.e., participants who ended the survey at the consent question) and break-offs (i.e., participants who were 18 years or older but did not complete an NCD module). The response rate was calculated as the number of complete and partial interviews divided by the total number of complete and partial interviews, refusals, break-offs, and the estimated proportion of age-eligible (e) unknowns (U). Unknown (U) participants were individuals who initiated the survey but did not answer the age question. The estimated proportion of unknown eligibility (e) was obtained from the 'proportion of participants' who selected the age-screen question as 18 years or older. Participants were classified as ineligible if they indicated an age below 18 years or did not pick up the phone. As a secondary analysis, contact refusal and response rates were calculated without applying e for the unknown participants."

6a-i) Online questionnaires: describe if they were validated for online use and apply CHERRIES items to describe how the questionnaires were designed/deployed

If outcomes were obtained through online questionnaires, describe if they were validated for online use and apply CHERRIES items to describe how the questionnaires were designed/deployed [9].

	1	2	3	4	5	
subitem not at all important	◉	○	○	○	○	essential

Clear selection

Does your paper address subitem 6a-i?

Copy and paste relevant sections from manuscript text

Not applicable to this study

6a-ii) Describe whether and how "use" (including intensity of use/dosage) was defined/measured/monitored

Describe whether and how "use" (including intensity of use/dosage) was defined/measured/monitored (logins, logfile analysis, etc.). Use/adoption metrics are important process outcomes that should be reported in any ehealth trial.

	1	2	3	4	5	
subitem not at all important	◉	○	○	○	○	essential

Clear selection

Does your paper address subitem 6a-ii?

Copy and paste relevant sections from manuscript text

Not applicable to this study

6a-iii) Describe whether, how, and when qualitative feedback from participants was obtained

Describe whether, how, and when qualitative feedback from participants was obtained (e.g., through emails, feedback forms, interviews, focus groups).

	1	2	3	4	5	
subitem not at all important	(●)	( )	( )	( )	( )	essential

Clear selection

Does your paper address subitem 6a-iii?

Copy and paste relevant sections from manuscript text

Not applicable to this study

6b) Any changes to trial outcomes after the trial commenced, with reasons

Does your paper address CONSORT subitem 6b? *

Copy and paste relevant sections from the manuscript (include quotes in quotation marks "like this" to indicate direct quotes from your manuscript), or elaborate on this item by providing additional information not in the ms, or briefly explain why the item is not applicable/relevant for your study

Not applicable to this study

7a) How sample size was determined

NPT: When applicable, details of whether and how the clustering by care provides or centers was addressed

7a-i) Describe whether and how expected attrition was taken into account when calculating the sample size

Describe whether and how expected attrition was taken into account when calculating the sample size.

	1	2	3	4	5	
subitem not at all important	○	○	○	○	◉	essential

Clear selection

Does your paper address subitem 7a-i?

Copy and paste relevant sections from manuscript title (include quotes in quotation marks "like this" to indicate direct quotes from your manuscript), or elaborate on this item by providing additional information not in the ms, or briefly explain why the item is not applicable/relevant for your study

"We used the same assumptions to calculate required sample sizes for the trial in both countries. With a 30% cooperation rate of the control arm, 5% type 1 error and 80% power, 376 participants were required to complete the interview for each study arm in order to detect a 10% difference between control and incentive groups. The total required sample size (i.e., complete surveys) was 1128 in each country. As recommended by the Rothman, we did not inflate the sample for multiple comparisons [34]. "

7b) When applicable, explanation of any interim analyses and stopping guidelines

Does your paper address CONSORT subitem 7b? *

Copy and paste relevant sections from the manuscript (include quotes in quotation marks "like this" to indicate direct quotes from your manuscript), or elaborate on this item by providing additional information not in the ms, or briefly explain why the item is not applicable/relevant for your study

Not applicable to this study

8a) Method used to generate the random allocation sequence

NPT: When applicable, how care providers were allocated to each trial group

Does your paper address CONSORT subitem 8a? *

Copy and paste relevant sections from the manuscript (include quotes in quotation marks "like this" to indicate direct quotes from your manuscript), or elaborate on this item by providing additional information not in the ms, or briefly explain why the item is not applicable/relevant for your study

"Participants were sampled using a fully-automated random digit dialing (RDD) method [27]. Briefly, the country codes along with the three digit-sequence specific to the mobile network operators were used as the base for potential mobile phone numbers. The remaining seven digits were then generated randomly. Respondents who self-reported being aged 18 years or older were eligible for the trial. The survey was programmed to have a designated local number appear on the respondent's caller ID screen."

8b) Type of randomisation; details of any restriction (such as blocking and block size)

Does your paper address CONSORT subitem 8b? *

Copy and paste relevant sections from the manuscript (include quotes in quotation marks "like this" to indicate direct quotes from your manuscript), or elaborate on this item by providing additional information not in the ms, or briefly explain why the item is not applicable/relevant for your study

"In this trial, incentives were delivered as airtime (i.e., mobile phone balance). Participants were randomized to one of three study arms: 1) no incentive (control group); 2) a promised airtime incentive of 50 Bangladeshi Taka (BDT; $0.6 USD) or 5000 Ugandan Shilling (UGX; $1.35 USD) for completing the IVR survey; or 3) lottery incentive (500 BDT and 100,000 UGX) where the odds of winning the lottery were 1:20. The conduct, analysis, and reporting of results were done in accordance with the Consolidated Standards of Reporting Trials guidelines [26]. "

9) Mechanism used to implement the random allocation sequence (such as sequentially numbered containers), describing any steps taken to conceal the sequence until interventions were assigned

Does your paper address CONSORT subitem 9? *

Copy and paste relevant sections from the manuscript (include quotes in quotation marks "like this" to indicate direct quotes from your manuscript), or elaborate on this item by providing additional information not in the ms, or briefly explain why the item is not applicable/relevant for your study

"The automated randomization process was done within the IVR platform to cover all mobile phone networks in both countries. Participants were randomized after selecting the survey language, but prior to consent (Supporting Figure 1). Due to the nature of the study design, participants were informed about the incentive during the survey introduction and therefore not blinded to study arm allocation. Statisticians involved in data cleaning and analysis were blinded to participants' allocation group. "

10) Who generated the random allocation sequence, who enrolled participants, and who assigned participants to interventions

Does your paper address CONSORT subitem 10? *

Copy and paste relevant sections from the manuscript (include quotes in quotation marks "like this" to indicate direct quotes from your manuscript), or elaborate on this item by providing additional information not in the ms, or briefly explain why the item is not applicable/relevant for your study

"The automated randomization process was done within the IVR platform to cover all mobile phone networks in both countries. Participants were randomized after selecting the survey language, but prior to consent (Supporting Figure 1). Due to the nature of the study design, participants were informed about the incentive during the survey introduction and therefore not blinded to study arm allocation. Statisticians involved in data cleaning and analysis were blinded to participants' allocation group. "

11a) If done, who was blinded after assignment to interventions (for example, participants, care providers, those assessing outcomes) and how

NPT: Whether or not administering co-interventions were blinded to group assignment

11a-i) Specify who was blinded, and who wasn't

Specify who was blinded, and who wasn't. Usually, in web-based trials it is not possible to blind the participants [1, 3] (this should be clearly acknowledged), but it may be possible to blind outcome assessors, those doing data analysis or those administering co-interventions (if any).

	1	2	3	4	5	
subitem not at all important	○	○	○	○	◉	essential

Clear selection

Does your paper address subitem 11a-i? *

Copy and paste relevant sections from the manuscript (include quotes in quotation marks "like this" to indicate direct quotes from your manuscript), or elaborate on this item by providing additional information not in the ms, or briefly explain why the item is not applicable/relevant for your study

"Due to the nature of the study design, participants were informed about the incentive during the survey introduction and therefore not blinded to study arm allocation. Statisticians involved in data cleaning and analysis were blinded to participants' allocation group."

11a-ii) Discuss e.g., whether participants knew which intervention was the "intervention of interest" and which one was the "comparator"

Informed consent procedures (4a-ii) can create biases and certain expectations - discuss e.g., whether participants knew which intervention was the "intervention of interest" and which one was the "comparator".

	1	2	3	4	5	
subitem not at all important	◉	○	○	○	○	essential

Clear selection

Does your paper address subitem 11a-ii?

Copy and paste relevant sections from the manuscript (include quotes in quotation marks "like this" to indicate direct quotes from your manuscript), or elaborate on this item by providing additional information not in the ms, or briefly explain why the item is not applicable/relevant for your study

Not applicable to this study

11b) If relevant, description of the similarity of interventions

(this item is usually not relevant for ehealth trials as it refers to similarity of a placebo or sham intervention to a active medication/intervention)

Does your paper address CONSORT subitem 11b? *

Copy and paste relevant sections from the manuscript (include quotes in quotation marks "like this" to indicate direct quotes from your manuscript), or elaborate on this item by providing additional information not in the ms, or briefly explain why the item is not applicable/relevant for your study

Not applicable to this study

12a) Statistical methods used to compare groups for primary and secondary outcomes

NPT: When applicable, details of whether and how the clustering by care providers or centers was addressed

Does your paper address CONSORT subitem 12a? *

Copy and paste relevant sections from the manuscript (include quotes in quotation marks "like this" to indicate direct quotes from your manuscript), or elaborate on this item by providing additional information not in the ms, or briefly explain why the item is not applicable/relevant for your study

"Demographic characteristics of complete interviews were described by study arms and compared using chi-square tests. Using the control arm as the reference category, risk ratios (RR) and 95% confidence intervals (CI) for the RRs were obtained for contact, response, refusal, and cooperation rates with log-binomial regression [32]. To assess any potential effect modification of incentives on cooperation rates, the log-binomial models were extended and interaction terms with education, gender, age, and location, were tested. We did not assess any effect modification for response rates because its equation includes Unknown disposition codes; which are participants who did not answer any of the demographic questions.

We calculated pooled risk ratios for the different incentive groups using DerSimonian and Laird random-effects meta-analysis [33]. The heterogeneity statistic (i.e., I-squared) was estimated by the Mantel-Haenszel method. The I-squared statistic indicates the proportion of variability in effect that resulted from heterogeneity instead of chance or sampling error. A lower value of I-squared statistic suggests lower heterogenicity. We calculated the direct delivery cost per complete survey, which included the cost of airtime used to deliver the survey and the incentive amount, as applicable. We summed the total call durations by arm and multiplied by the per-minute cost of airtime – $0.04 USD in Bangladesh and $0.10 USD in Uganda – to produce the estimated cost per completed survey. Stata 14.0 was used to analyze data (Stata Corp, College Station, TX, USA). An alpha of 0.05 was assumed for all tests of statistical significance."

12a-i) Imputation techniques to deal with attrition / missing values

Imputation techniques to deal with attrition / missing values: Not all participants will use the intervention/comparator as intended and attrition is typically high in ehealth trials. Specify how participants who did not use the application or dropped out from the trial were treated in the statistical analysis (a complete case analysis is strongly discouraged, and simple imputation techniques such as LOCF may also be problematic [4]).

	1	2	3	4	5	
subitem not at all important	(●)	( )	( )	( )	( )	essential

Clear selection

Does your paper address subitem 12a-i? *

Copy and paste relevant sections from the manuscript (include quotes in quotation marks "like this" to indicate direct quotes from your manuscript), or elaborate on this item by providing additional information not in the ms, or briefly explain why the item is not applicable/relevant for your study

Not applicable to this study

12b) Methods for additional analyses, such as subgroup analyses and adjusted analyses

Does your paper address CONSORT subitem 12b? *

Copy and paste relevant sections from the manuscript (include quotes in quotation marks "like this" to indicate direct quotes from your manuscript), or elaborate on this item by providing additional information not in the ms, or briefly explain why the item is not applicable/relevant for your study

"We did not assess any effect modification for response rates because its equation includes Unknown disposition codes; which are participants who did not answer any of the demographic questions."

X26) REB/IRB Approval and Ethical Considerations [recommended as subheading under "Methods"] (not a CONSORT item)

X26-i) Comment on ethics committee approval

	1	2	3	4	5	
subitem not at all important	○	○	○	○	◉	essential

Clear selection

Does your paper address subitem X26-i?

Copy and paste relevant sections from the manuscript (include quotes in quotation marks "like this" to indicate direct quotes from your manuscript), or elaborate on this item by providing additional information not in the ms, or briefly explain why the item is not applicable/relevant for your study

"Review boards of the following institutions approved the study protocol: 1) Johns Hopkins Bloomberg School of Public Health, Baltimore, MD, USA; 2) Makerere University School of Public Health, Kampala, Uganda; 3) The Uganda National Council for Science and Technology, Kampala, Uganda; and 4) The Institute of Epidemiology Disease Control and Research (IEDCR), Dhaka, Bangladesh. A detailed description of the study protocol has been published elsewhere [30]. The ClinicalTrials.gov registration number of the study is NCT03773146."

x26-ii) Outline informed consent procedures

Outline informed consent procedures e.g., if consent was obtained offline or online (how? Checkbox, etc.?), and what information was provided (see 4a-ii). See [6] for some items to be included in informed consent documents.

	1	2	3	4	5	
subitem not at all important	○	○	○	○	◉	essential

Clear selection

Does your paper address subitem X26-ii?

Copy and paste relevant sections from the manuscript (include quotes in quotation marks "like this" to indicate direct quotes from your manuscript), or elaborate on this item by providing additional information not in the ms, or briefly explain why the item is not applicable/relevant for your study

"Respondents who picked up the phone were instructed to select a language from a list of languages; Bangla or English in Bangladesh and Luganda, Luo, Runyakitara, or English in Uganda. Then, participants listened to a survey introduction where the survey objectives and requirements for the incentive (i.e., survey completion), as applicable, were described (Supporting Table 1 and Supporting Figure 1). Participants were told that they would not incur any expenses by answering the survey. Then, age-eligibility was confirmed (i.e., "Are you 18 years or older? If Yes, Press 1. If No, Press 3"). Age-eligible participants were provided the consent disclosure statement and requested to authorize their participation by pressing the 1 button on the mobile phone. Next, they answered demographic and NCD questions. Only those who completed the survey received the incentive. Participants were instructed to press the 'star' key to repeat any questions. "

X26-iii) Safety and security procedures

Safety and security procedures, incl. privacy considerations, and any steps taken to reduce the likelihood or detection of harm (e.g., education and training, availability of a hotline)

	1	2	3	4	5	
subitem not at all important	○	○	○	○	◉	essential

Clear selection

Does your paper address subitem X26-iii?

Copy and paste relevant sections from the manuscript (include quotes in quotation marks "like this" to indicate direct quotes from your manuscript), or elaborate on this item by providing additional information not in the ms, or briefly explain why the item is not applicable/relevant for your study

"Respondents who picked up the phone were instructed to select a language from a list of languages; Bangla or English in Bangladesh and Luganda, Luo, Runyakitara, or English in Uganda. Then, participants listened to a survey introduction where the survey objectives and requirements for the incentive (i.e., survey completion), as applicable, were described (Supporting Table 1 and Supporting Figure 1). Participants were told that they would not incur any expenses by answering the survey. Then, age-eligibility was confirmed (i.e., "Are you 18 years or older? If Yes, Press 1. If No, Press 3"). Age-eligible participants were provided the consent disclosure statement and requested to authorize their participation by pressing the 1 button on the mobile phone. Next, they answered demographic and NCD questions. Only those who completed the survey received the incentive. Participants were instructed to press the 'star' key to repeat any questions."

RESULTS

13a) For each group, the numbers of participants who were randomly assigned, received intended treatment, and were analysed for the primary outcome

NPT: The number of care providers or centers performing the intervention in each group and the number of patients treated by each care provider in each center

Does your paper address CONSORT subitem 13a? *

Copy and paste relevant sections from the manuscript (include quotes in quotation marks "like this" to indicate direct quotes from your manuscript), or elaborate on this item by providing additional information not in the ms, or briefly explain why the item is not applicable/relevant for your study

Yes, Figures 1 and 2 also show that "From June 14, 2017 to July 14, 2017, a total of 1165 compete interviews were obtained from 546,746 RDD phone calls in Bangladesh (Fig 1). In Uganda, 178,572 RDD calls were made between March 26 and April 22, 2017 to obtain 1,248 complete interviews (Fig 2). "

13b) For each group, losses and exclusions after randomisation, together with reasons

Does your paper address CONSORT subitem 13b? (NOTE: Preferably, this is shown in a CONSORT flow diagram) *

Copy and paste relevant sections from the manuscript (include quotes in quotation marks "like this" to indicate direct quotes from your manuscript), or elaborate on this item by providing additional information not in the ms, or briefly explain why the item is not applicable/relevant for your study

"From June 14, 2017 to July 14, 2017, a total of 1165 compete interviews were obtained from 546,746 RDD phone calls in Bangladesh (Fig 1). In Uganda, 178,572 RDD calls were made between March 26 and April 22, 2017 to obtain 1,248 complete interviews (Fig 2). In both countries, the sociodemographic characteristics of complete interviews were similar across study arms (Table 1). Overall, 89.4% (n= 1,042) of Bangladeshi respondents were males while 76.0% (n=948) Ugandan respondents were males. Most respondents were between the ages of 18 to 29 years old, 74.4% (n=867), and 71.0% (n=886), in Bangladesh and Uganda, respectively. "

13b-i) Attrition diagram

Strongly recommended: An attrition diagram (e.g., proportion of participants still logging in or using the intervention/comparator in each group plotted over time, similar to a survival curve) or other figures or tables demonstrating usage/dose/engagement.

	1	2	3	4	5	
subitem not at all important	○	○	○	○	◉	essential

Clear selection

Does your paper address subitem 13b-i?

Copy and paste relevant sections from the manuscript or cite the figure number if applicable (include quotes in quotation marks "like this" to indicate direct quotes from your manuscript), or elaborate on this item by providing additional information not in the ms, or briefly explain why the item is not applicable/relevant for your study

Yes, Figures 1 and 2 show it

14a) Dates defining the periods of recruitment and follow-up

Does your paper address CONSORT subitem 14a? *

Copy and paste relevant sections from the manuscript (include quotes in quotation marks "like this" to indicate direct quotes from your manuscript), or elaborate on this item by providing additional information not in the ms, or briefly explain why the item is not applicable/relevant for your study

"From June 14, 2017 to July 14, 2017, a total of 1165 compete interviews were obtained from 546,746 RDD phone calls in Bangladesh (Fig 1). In Uganda, 178,572 RDD calls were made between March 26 and April 22, 2017 to obtain 1,248 complete interviews (Fig 2). "

14a-i) Indicate if critical “secular events” fell into the study period

Indicate if critical “secular events” fell into the study period, e.g., significant changes in Internet resources available or “changes in computer hardware or Internet delivery resources”

	1	2	3	4	5	
subitem not at all important	(●)	○	○	○	○	essential

Clear selection

Does your paper address subitem 14a-i?

Copy and paste relevant sections from the manuscript (include quotes in quotation marks "like this" to indicate direct quotes from your manuscript), or elaborate on this item by providing additional information not in the ms, or briefly explain why the item is not applicable/relevant for your study

Not applicable to this study

14b) Why the trial ended or was stopped (early)

Does your paper address CONSORT subitem 14b? *

Copy and paste relevant sections from the manuscript (include quotes in quotation marks "like this" to indicate direct quotes from your manuscript), or elaborate on this item by providing additional information not in the ms, or briefly explain why the item is not applicable/relevant for your study

Not applicable to this study

15) A table showing baseline demographic and clinical characteristics for each group

NPT: When applicable, a description of care providers (case volume, qualification, expertise, etc.) and centers (volume) in each group

Does your paper address CONSORT subitem 15? *

Copy and paste relevant sections from the manuscript (include quotes in quotation marks "like this" to indicate direct quotes from your manuscript), or elaborate on this item by providing additional information not in the ms, or briefly explain why the item is not applicable/relevant for your study

Yes, Table 1 shows demographic characteristics

15-i) Report demographics associated with digital divide issues

In ehealth trials it is particularly important to report demographics associated with digital divide issues, such as age, education, gender, social-economic status, computer/Internet/ehealth literacy of the participants, if known.

	1	2	3	4	5	
subitem not at all important	(●)	( )	( )	( )	( )	essential

Clear selection

Does your paper address subitem 15-i? *

Copy and paste relevant sections from the manuscript (include quotes in quotation marks "like this" to indicate direct quotes from your manuscript), or elaborate on this item by providing additional information not in the ms, or briefly explain why the item is not applicable/relevant for your study

Yes, this was only among people who can answer questions using phones

16) For each group, number of participants (denominator) included in each analysis and whether the analysis was by original assigned groups

16-i) Report multiple “denominators” and provide definitions

Report multiple “denominators” and provide definitions: Report N’s (and effect sizes) “across a range of study participation [and use] thresholds” [1], e.g., N exposed, N consented, N used more than x times, N used more than y weeks, N participants “used” the intervention/comparator at specific pre-defined time points of interest (in absolute and relative numbers per group). Always clearly define “use” of the intervention.

	1	2	3	4	5	
subitem not at all important	○	○	○	○	◉	essential

Clear selection

Does your paper address subitem 16-i? *

Copy and paste relevant sections from the manuscript (include quotes in quotation marks "like this" to indicate direct quotes from your manuscript), or elaborate on this item by providing additional information not in the ms, or briefly explain why the item is not applicable/relevant for your study

Yes, "Table 2 also shows the disposition codes and survey rates in both countries. In Bangladesh, the cooperation and response rates were, respectively, 28.4% (393/1383) and 21.0% (675/3216), in the control arm, 39.3% (413/1051) and 26.5% (588/2222) in the promised incentive arm, and 36.6% (359/980) and 24.5% (544/2220) in lottery incentive arm. Cooperation and response rates were significantly higher in the promised incentive arm, respectively, (RR: 1.38, 1.24–1.55, p<.001) and (RR: 1.26, 95% CI: 1.14–1.39, p<.001), and in the lottery arm, (RR: 1.28, 95% CI: 1.15–1.45, p<.001) and (RR: 1.17, 95% CI: 1.06–1.29, p=.002), as compared to the control arm. In Uganda, the cooperation and response rates were higher than Bangladesh. Similar to Bangladesh, cooperation and response rates, respectively, were significantly higher in the promised, (RR: 1.47, 95% CI: 1.33–1.62, p<.001) and (RR: 1.27, 95% CI: 1.16–1.39, p<.001), and lottery arm, (RR: 1.34, 95% CI: 1.21–1.48, p<.001) and (RR: 1.17, 95% CI: 1.06–1.29, p=.001), as compared to control arm. In both countries, cooperation and response rates were similar when using equations that do not include the estimated proportion of age-eligible participants in the unknown disposition code (see Supporting Table 4). In both countries, sub-group analyses found that participant gender, age, education, and location did not modify the intervention's effect on cooperation rate (Supporting Tables 5 and 6). "

16-ii) Primary analysis should be intent-to-treat

Primary analysis should be intent-to-treat, secondary analyses could include comparing only "users", with the appropriate caveats that this is no longer a randomized sample (see 18-i).

	1	2	3	4	5	
subitem not at all important	○	○	○	○	◉	essential

Clear selection

Does your paper address subitem 16-ii?

Copy and paste relevant sections from the manuscript (include quotes in quotation marks "like this" to indicate direct quotes from your manuscript), or elaborate on this item by providing additional information not in the ms, or briefly explain why the item is not applicable/relevant for your study

The analysis was wholly intent to treat, there was no scope of analysis without ITT

17a) For each primary and secondary outcome, results for each group, and the estimated effect size and its precision (such as 95% confidence interval)

Does your paper address CONSORT subitem 17a? *

Copy and paste relevant sections from the manuscript (include quotes in quotation marks "like this" to indicate direct quotes from your manuscript), or elaborate on this item by providing additional information not in the ms, or briefly explain why the item is not applicable/relevant for your study

"Table 2 also shows the disposition codes and survey rates in both countries. In Bangladesh, the cooperation and response rates were, respectively, 28.4% (393/1383) and 21.0% (675/3216), in the control arm, 39.3% (413/1051) and 26.5% (588/2222) in the promised incentive arm, and 36.6% (359/980) and 24.5% (544/2220) in lottery incentive arm. Cooperation and response rates were significantly higher in the promised incentive arm, respectively, (RR: 1.38, 1.24–1.55, p<.001) and (RR: 1.26, 95% CI: 1.14–1.39, p<.001), and in the lottery arm, (RR: 1.28, 95% CI: 1.15–1.45, p<.001) and (RR: 1.17, 95% CI: 1.06–1.29, p=.002), as compared to the control arm. In Uganda, the cooperation and response rates were higher than Bangladesh. Similar to Bangladesh, cooperation and response rates, respectively, were significantly higher in the promised, (RR: 1.47, 95% CI: 1.33–1.62, p<.001) and (RR: 1.27, 95% CI: 1.16–1.39, p<.001), and lottery arm, (RR: 1.34, 95% CI: 1.21–1.48, p<.001) and (RR: 1.17, 95% CI: 1.06–1.29, p=.001), as compared to control arm. In both countries, cooperation and response rates were similar when using equations that do not include the estimated proportion of age-eligible participants in the unknown disposition code (see Supporting Table 4). In both countries, sub-group analyses found that participant gender, age, education, and location did not modify the intervention's effect on cooperation rate (Supporting Tables 5 and 6).
The pooling of Bangladesh and Uganda participants found that the promised incentive (pooled RR (pRR): 1.42, 95%CI 1.32, 1.53) and lottery incentive (pRR: 1.31, 95%CI 1.21, 1.41) significantly improved cooperation rate as compared to those not receiving an incentive (Figure 3). Similarly, the response rate was significantly higher in the promised incentive (pRR: 1.26, 95%CI 1.18, 1.35) and lottery incentive (pRR: 1.17, 1.09, 1.25; I-squared 0.0%) as compared to controls. Overall, providing any incentive significantly improved cooperation and response rate by 37% (pRR 1.37, 95%CI 1.29, 1.44) and 22% (pRR: 1.22 95%CI 1.18, 1.28) and these results were highly consistent, (I-squared 12.1%, p = .33) and (I-squared 0.0%, p = .47).
"

17a-i) Presentation of process outcomes such as metrics of use and intensity of use

In addition to primary/secondary (clinical) outcomes, the presentation of process outcomes such as metrics of use and intensity of use (dose, exposure) and their operational definitions is critical. This does not only refer to metrics of attrition (13-b) (often a binary variable), but also to more continuous exposure metrics such as “average session length”. These must be accompanied by a technical description how a metric like a “session” is defined (e.g., timeout after idle time) [1] (report under item 6a).

	1	2	3	4	5	
subitem not at all important	◉	○	○	○	○	essential

Clear selection

Does your paper address subitem 17a-i?

Copy and paste relevant sections from the manuscript (include quotes in quotation marks "like this" to indicate direct quotes from your manuscript), or elaborate on this item by providing additional information not in the ms, or briefly explain why the item is not applicable/relevant for your study

Not applicable to the study

17b) For binary outcomes, presentation of both absolute and relative effect sizes is recommended

Does your paper address CONSORT subitem 17b? *

Copy and paste relevant sections from the manuscript (include quotes in quotation marks "like this" to indicate direct quotes from your manuscript), or elaborate on this item by providing additional information not in the ms, or briefly explain why the item is not applicable/relevant for your study

Not applicable to the study

18) Results of any other analyses performed, including subgroup analyses and adjusted analyses, distinguishing pre-specified from exploratory

Does your paper address CONSORT subitem 18? *

Copy and paste relevant sections from the manuscript (include quotes in quotation marks "like this" to indicate direct quotes from your manuscript), or elaborate on this item by providing additional information not in the ms, or briefly explain why the item is not applicable/relevant for your study

Not applicable to the study

18-i) Subgroup analysis of comparing only users

A subgroup analysis of comparing only users is not uncommon in ehealth trials, but if done, it must be stressed that this is a self-selected sample and no longer an unbiased sample from a randomized trial (see 16-iii).

	1	2	3	4	5	
subitem not at all important	◉	○	○	○	○	essential

Clear selection

Does your paper address subitem 18-i?

Copy and paste relevant sections from the manuscript (include quotes in quotation marks "like this" to indicate direct quotes from your manuscript), or elaborate on this item by providing additional information not in the ms, or briefly explain why the item is not applicable/relevant for your study

Not applicable to the study

19) All important harms or unintended effects in each group

(for specific guidance see CONSORT for harms)

Does your paper address CONSORT subitem 19? *

Copy and paste relevant sections from the manuscript (include quotes in quotation marks "like this" to indicate direct quotes from your manuscript), or elaborate on this item by providing additional information not in the ms, or briefly explain why the item is not applicable/relevant for your study

Not applicable to the study

19-i) Include privacy breaches, technical problems

Include privacy breaches, technical problems. This does not only include physical “harm” to participants, but also incidents such as perceived or real privacy breaches [1], technical problems, and other unexpected/unintended incidents. “Unintended effects” also includes unintended positive effects [2].

	1	2	3	4	5	
subitem not at all important	(•)	( )	( )	( )	( )	essential

Clear selection

Does your paper address subitem 19-i?

Copy and paste relevant sections from the manuscript (include quotes in quotation marks "like this" to indicate direct quotes from your manuscript), or elaborate on this item by providing additional information not in the ms, or briefly explain why the item is not applicable/relevant for your study

Not applicable to the study

19-ii) Include qualitative feedback from participants or observations from staff/researchers

Include qualitative feedback from participants or observations from staff/researchers, if available, on strengths and shortcomings of the application, especially if they point to unintended/unexpected effects or uses. This includes (if available) reasons for why people did or did not use the application as intended by the developers.

	1	2	3	4	5	
subitem not at all important	(•)	( )	( )	( )	( )	essential

Clear selection

Does your paper address subitem 19-ii?

Copy and paste relevant sections from the manuscript (include quotes in quotation marks "like this" to indicate direct quotes from your manuscript), or elaborate on this item by providing additional information not in the ms, or briefly explain why the item is not applicable/relevant for your study

Not applicable to the study

DISCUSSION

22) Interpretation consistent with results, balancing benefits and harms, and considering other relevant evidence

NPT: In addition, take into account the choice of the comparator, lack of or partial blinding, and unequal expertise of care providers or centers in each group

22-i) Restate study questions and summarize the answers suggested by the data, starting with primary outcomes and process outcomes (use)

Restate study questions and summarize the answers suggested by the data, starting with primary outcomes and process outcomes (use).

	1	2	3	4	5	
subitem not at all important	○	○	○	○	◉	essential

Clear selection

Does your paper address subitem 22-i? *

Copy and paste relevant sections from the manuscript (include quotes in quotation marks "like this" to indicate direct quotes from your manuscript), or elaborate on this item by providing additional information not in the ms, or briefly explain why the item is not applicable/relevant for your study

Yes, "In this study, the promised and lottery incentive arms had significantly higher response and cooperation rates than control arms (i.e., no incentive group) in both Bangladesh and Uganda. Given that the pooled analysis found that the incentive's effect was highly consistent at increasing these survey rates in two culturally and geographically distinct LMICs, the provision of airtime incentives may be a useful mechanism to increase IVR survey participation in other low-resource settings. "

22-ii) Highlight unanswered new questions, suggest future research

Highlight unanswered new questions, suggest future research.

	1	2	3	4	5	
subitem not at all important	○	○	○	○	◉	essential

Clear selection

Does your paper address subitem 22-ii?

Copy and paste relevant sections from the manuscript (include quotes in quotation marks "like this" to indicate direct quotes from your manuscript), or elaborate on this item by providing additional information not in the ms, or briefly explain why the item is not applicable/relevant for your study

"We observed a higher proportion of male, young (i.e., 18-29-year-olds), or urban residents compared to general population in both countries. This finding was similar to RDD IVR surveys conducted in Afghanistan, Ethiopia, Ghana, Mozambique, Tanzania, and Zimbabwe [38,45,46]. Male gender, younger age, higher education, and urban residence have been found to be associated with mobile phone ownership in LMICs, including Bangladesh and East Africa [47,48]. This does raise concerns about the ability to generate nationally representative estimates (i.e., generalizability of the findings). Advances in sampling and statistical methodology may be required for such estimates. Quota sampling could be used to ensure a more equal distribution of the sociodemographic characteristics [49]. Others have found that weighted estimates of NCD indicators collected via MPS approximate household collected data [50]. "

20) Trial limitations, addressing sources of potential bias, imprecision, and, if relevant, multiplicity of analyses

20-i) Typical limitations in ehealth trials

Typical limitations in ehealth trials: Participants in ehealth trials are rarely blinded. Ehealth trials often look at a multiplicity of outcomes, increasing risk for a Type I error. Discuss biases due to non-use of the intervention/usability issues, biases through informed consent procedures, unexpected events.

	1	2	3	4	5	
subitem not at all important	○	○	○	○	◉	essential

Clear selection

Does your paper address subitem 20-i? *

Copy and paste relevant sections from the manuscript (include quotes in quotation marks "like this" to indicate direct quotes from your manuscript), or elaborate on this item by providing additional information not in the ms, or briefly explain why the item is not applicable/relevant for your study

"In addition to underrepresentation from some sociodemographic populations, this study has some limitations. First, there was a substantial number of phone calls in Bangladesh and Uganda where we were unable to determine the status of the phone number. Calling people randomly can also reduce the response. We could not determine if it was a working number or a non-working number [51]. As randomization to study arm occurred after participants picked up the phone, we chose to designate these phone calls as non-working numbers. This decision inflates our contact, response, and refusal rates, but has no effect on the cooperation rate. Second, although not an issue in Bangladesh where 99% of the respondents took the IVR survey in Bangla, our survey was only available in three of the six major language groups in Uganda [52]. This might lead to some selection error due to unavailability of the preferred language and would have larger implications for nationally representative surveys [46]. We did not check about the quality of collected data as that was not the main purpose of this study; future studies should investigate that. "

21) Generalisability (external validity, applicability) of the trial findings

NPT: External validity of the trial findings according to the intervention, comparators, patients, and care providers or centers involved in the trial

21-i) Generalizability to other populations

Generalizability to other populations: In particular, discuss generalizability to a general Internet population, outside of a RCT setting, and general patient population, including applicability of the study results for other organizations

	1	2	3	4	5	
subitem not at all important	○	○	○	◉	○	essential

Clear selection

Does your paper address subitem 21-i?

Copy and paste relevant sections from the manuscript (include quotes in quotation marks "like this" to indicate direct quotes from your manuscript), or elaborate on this item by providing additional information not in the ms, or briefly explain why the item is not applicable/relevant for your study

"First, the randomization was automated and embedded within the IVR platform. This safeguarded against misallocation of participants to study arm which could bias response and cooperation rates. Second, we employed standardized protocols and questionnaires in both countries and used the same technology platform to deliver IVR surveys to afford for cross-country comparisons. Lastly, our sampling frame consisted of all known mobile network operators in each country; thereby minimizing potential selection bias."

21-ii) Discuss if there were elements in the RCT that would be different in a routine application setting

Discuss if there were elements in the RCT that would be different in a routine application setting (e.g., prompts/reminders, more human involvement, training sessions or other co-interventions) and what impact the omission of these elements could have on use, adoption, or outcomes if the intervention is applied outside of a RCT setting.

	1	2	3	4	5	
subitem not at all important	◉	○	○	○	○	essential

Clear selection

Does your paper address subitem 21-ii?

Copy and paste relevant sections from the manuscript (include quotes in quotation marks "like this" to indicate direct quotes from your manuscript), or elaborate on this item by providing additional information not in the ms, or briefly explain why the item is not applicable/relevant for your study

Not applicable to this study

OTHER INFORMATION

23) Registration number and name of trial registry

Does your paper address CONSORT subitem 23? *

Copy and paste relevant sections from the manuscript (include quotes in quotation marks "like this" to indicate direct quotes from your manuscript), or elaborate on this item by providing additional information not in the ms, or briefly explain why the item is not applicable/relevant for your study

Yes, "The ClinicalTrials.gov registration number of the study is NCT03773146."

24) Where the full trial protocol can be accessed, if available

Does your paper address CONSORT subitem 24? *

Cite a Multimedia Appendix, other reference, or copy and paste relevant sections from the manuscript (include quotes in quotation marks "like this" to indicate direct quotes from your manuscript), or elaborate on this item by providing additional information not in the ms, or briefly explain why the item is not applicable/relevant for your study

Yes, "Gibson DG, Pariyo GW, Wosu AC, Greenleaf AR, Ali J, Ahmed S, Labrique AB, Islam K, Masanja H, Rutebemberwa E, Hyder AA. Evaluation of Mechanisms to Improve Performance of Mobile Phone Surveys in Low- and Middle-Income Countries: Research Protocol. JMIR Res Protoc 2017;6(5):e81. [doi: 10.2196/resprot.7534]"

25) Sources of funding and other support (such as supply of drugs), role of funders

Does your paper address CONSORT subitem 25? *

Copy and paste relevant sections from the manuscript (include quotes in quotation marks "like this" to indicate direct quotes from your manuscript), or elaborate on this item by providing additional information not in the ms, or briefly explain why the item is not applicable/relevant for your study

"This study was made possible by the generous support of Bloomberg Philanthropies (https://www.bloomberg.org/) grant no. 41388.01 to AAH and the people of Australia through the Department of Foreign Affairs and Trade (https://dfat.gov.au/pages/default.aspx). The contents are the responsibility of the authors and do not necessarily reflect the views of Bloomberg Philanthropies or the Government of Australia. The funders had no role in study design, data collection and analysis, decision to publish, or preparation of the manuscript. Competing interests: The authors have declared that no competing interests exist. "

X27) Conflicts of Interest (not a CONSORT item)

X27-i) State the relation of the study team towards the system being evaluated

In addition to the usual declaration of interests (financial or otherwise), also state the relation of the study team towards the system being evaluated, i.e., state if the authors/evaluators are distinct from or identical with the developers/sponsors of the intervention.

	1	2	3	4	5	
subitem not at all important	○	○	○	◉	○	essential

Clear selection

Does your paper address subitem X27-i?

Copy and paste relevant sections from the manuscript (include quotes in quotation marks "like this" to indicate direct quotes from your manuscript), or elaborate on this item by providing additional information not in the ms, or briefly explain why the item is not applicable/relevant for your study

"The authors declare that they have no competing interest."

About the CONSORT EHEALTH checklist

As a result of using this checklist, did you make changes in your manuscript? *

- ○ yes, major changes
- ○ yes, minor changes
- ◉ no

What were the most important changes you made as a result of using this checklist?

Not applicable to this paper

How much time did you spend on going through the checklist INCLUDING making changes in your manuscript *

2 hours of time required to do it

As a result of using this checklist, do you think your manuscript has improved? *

- (selected) yes
- no
- Other:

Would you like to become involved in the CONSORT EHEALTH group?

This would involve for example becoming involved in participating in a workshop and writing an "Explanation and Elaboration" document

- yes
- (selected) no
- Other:

Clear selection

Any other comments or questions on CONSORT EHEALTH

Not applicable, no comments about this

STOP - Save this form as PDF before you click submit

To generate a record that you filled in this form, we recommend to generate a PDF of this page (on a Mac, simply select "print" and then select "print as PDF") before you submit it.

When you submit your (revised) paper to JMIR, please upload the PDF as supplementary file.

Don't worry if some text in the textboxes is cut off, as we still have the complete information in our database. Thank you!

Final step: Click submit !

Click submit so we have your answers in our database!

Submit Clear form

Never submit passwords through Google Forms.

This content is neither created nor endorsed by Google. Report Abuse - Terms of Service - Privacy Policy

Google Forms
